# A generalized strain approach to anisotropic elasticity

**DOI:** 10.1038/s41598-021-03842-3

**Published:** 2022-01-07

**Authors:** M. H. B. M. Shariff

**Affiliations:** Department of Applied Mathematics and Science, Khalifa University of Science and Technology, Khalifa, UAE

**Keywords:** Engineering, Materials science, Physics

## Abstract

This work proposes a generalized Lagrangian strain function $f_\alpha$ (that depends on modified stretches) and a volumetric strain function $g_\alpha$ (that depends on the determinant of the deformation tensor) to characterize isotropic/anisotropic strain energy functions. With the aid of a spectral approach, the single-variable strain functions enable the development of strain energy functions that are consistent with their infinitesimal counterparts, including the development of a strain energy function for the general anisotropic material that contains the general 4th order classical stiffness tensor. The generality of the single-variable strain functions sets a platform for future development of adequate specific forms of the isotropic/anisotropic strain energy function; future modellers only require to construct specific forms of the functions $f_\alpha$ and $g_\alpha$ to model their strain energy functions. The spectral invariants used in the constitutive equation have a clear physical interpretation, which is attractive, in aiding experiment design and the construction of specific forms of the strain energy. Some previous strain energy functions that appeared in the literature can be considered as special cases of the proposed generalized strain energy function. The resulting constitutive equations can be easily converted, to allow the mechanical influence of compressed fibres to be excluded or partial excluded and to model fibre dispersion in collagenous soft tissues. Implementation of the constitutive equations in Finite Element software is discussed. The suggested crude specific strain function forms are able to fit the theory well with experimental data and managed to predict several sets of experimental data.

## Introduction

Hill^1^ introduced a generalized strain function in finite elasticity. Using a Hill’s strain function of the form

1 $$\begin{aligned} {\displaystyle \frac{\lambda ^\kappa -1}{\kappa }} \, , \end{aligned}$$

where $\kappa$ is real parameter and $\lambda$ is a principal stretch, Ogden^2^ successfully model the mechanical behaviour of incompressible isotropic solids. In the literature, different values of the parameter $\kappa$ are used to model different types of incompressible isotropic elastic solids; this suggests that the selection of strain functions to model a constitutive equation depends on the type of material we intend to model. Several special forms of the Hill’s generalized strain function used in modelling anisotropic/isotropic elastic solids can be found, for example in references^3–7^. In general, Hill’s strain invariants do not depend explicitly on right Cauchy-Green tensor ${\varvec{C}}$ and their 1st and 2nd order derivatives with respect to ${\varvec{C}}$ can only be obtained via spectral derivative formulae that are recently developed (see, for example references^8–11^) and, in view of this, the author believes that anisotropic/isotropic strain energy functions that are characterized by Hill’s generalized strain functions (to the best of the author’s knowledge) do not exist in the literature. This motivates the author to develop infinitesimal-consistent anisotropic/isotropic finite strain energy functions that are based on the generalized Hill’s strain function and the development requires proposing modified Hill’s and volumetric strain functions; it also requires a spectral approach based on the author’s previous work on anisotropic spectral models (see, for example references^12–15^) that used spectral invariants with a clear physical meaning. The advantages of spectral invariants over classical invariants^16^ in constitutive modelling are described, for example, in Shariff and Merodio^17^, hence we will not elaborate them here. In the excellent work of references^18–22^, spectral invariants have also been used to construct an infinitesimal-consistent anisotropic/isotropic finite strain energy function via the WYPiWYG approach, where the energy function shape and the material data of the model are obtained solving the equilibrium equations of the different experiments. In future, there might be a possibility to connect our approach to the WYPiWYG approach. Using an approach similar to that given in references^8,9,15,23^, our proposed model may be extended to model dissipative materials such as those discussed in^24–26^. Our proposed model may also be possibly extended to model strain gradient materials (see, for example references^26,27^) via a similar approach to that of Soltadtos et al.^28^.

The number of independent spectral invariants in an irreducible/minimal integrity basis can be easily derived^29–32^. A classical irreducible/minimal integrity basis, for a highly anisotropic material, contains a numerous amount of classical invariants^16^ and, due to their unclear physical interpretation, it is not clear in the literature how to select an appropriate (or optimum) subset of classical invariants from an irreducible/minimal integrity basis to represent a strain energy function: In particular, most traditional invariant-based approach to hyperelasticity, which uses classical invariants, typically allows discretion to deem which invariants are necessary for inclusion in the strain energy function for a given model. Omission of invariants offers mathematically simplified models and reduced number of ground-state material constants required for calibration (Shariff^12,14^ has shown that some of the well known models in the literature do not contain all their ground-state constants). However, the discrimination in selection of invariants is often debated, and neglecting the influence of some invariants may result in an incomplete representation of the full range of mechanical response subjected to a continuum^14,33–35^. In this communication, we show that the construction of a strain energy function that uses a full set of spectral invariants that is consistent with infinitesimal theory can be easily done via the use of, modified Hill’s and volumetric strain functions: A discussion on the importance of a nonlinear (finite strain) strain energy function that must be consistent with infinitesimal theory can be found, for example in, Rosa et al.^22^ and Shariff^14^.

In some materials, the mechanical influence of compressed fibres is negligible or is different from stretched fibres and, in soft tissues, the influence of fibre dispersion could be relevant in modelling constitutive equations: In Appendices A and B (Supplementary information), we illustrate how the proposed strain energy functions can be easily amended to take account of these influences.

### Remark

Valanis and Landel^36^ strain energy function

2 $$\begin{aligned} {W}_{(v)} = \sum _{i=1}^3 {\bar{r}}(\lambda _i) \, , \end{aligned}$$

where $\lambda _i$s are principal stretches, play an important role in modelling incompressible isotropic solids. The function ${\bar{r}}$ is arbitrary and this set a platform for modelling specific types of incompressibe isotropic elastic solids. Numerous specific forms of ${\bar{r}}$, that are able to successfully model the mechanical behaviour of incompressible isotropic solids, have been proposed in the literature, see for example Ogden^2^ and Shariff^37^. The single variable function ${\bar{r}}$ depends on an invariant with a clear physical meaning and this makes the Valanis and Landel form experimentally attractive^17^. The Valanis and Landel form impels us to develop anisotropic constitutive equations, which depends on single variable *aribtrary* functions that will set a platform for future modelling of specific types of anisotropic elastic solids. Our constitutive equations are developed via generalized strain single-variable functions. We overtly emphasize that, in this paper, we are not particularly concerned in obtaining specific forms of the proposed generalized strain functions. A rigourous construction of specific forms such those found in references^2,14,37^ requires a lot of work and it is beyond the scope of this paper. As mentioned above, the generalized constitutive equations described here will set a platform (analogous to the “generalized” Valanis and Landel form for isotropic elastic solids) for future modelling of specific types of anisotropic elastic solids.

## Preliminaries

In this paper, summation convention is not used and all subscripts *i*,*j* and *k* take the values 1, 2, 3, unless stated otherwise. Vectors and tensors are written in lowercase and uppercase bold fonts, respectively. Only quasi-static deformations and time-independent fields are considered. The mechanical body forces are assumed to be negligible. The deformation gradient is denoted by ${\varvec{F}}$ and ${\varvec{C}}= {\varvec{F}}^T{\varvec{F}}= {\varvec{U}}^2$, respectively, where ${\varvec{U}}$ is the right stretch tensor.

## General strain energy function

A general strain energy function for an elastic solid can be expressed as

3 $$\begin{aligned} {W}_{(A)} = W_a({\varvec{U}}) \, . \end{aligned}$$

The facilitate the construction of an incompressible material, regarded as a material recovered from the corresponding compressible material by taking the incompressible limit^38^, we use the modified stretch tensor

4 $$\begin{aligned} {\varvec{U}}^* = J^{-\frac{1}{3}}{\varvec{U}}\, , {\quad }\det {\varvec{U}}^* = 1 \, , \end{aligned}$$

where $J = \det {\varvec{F}}> 0$ and $\det$ is the determinant of a tensor. Hence, we express

5 $$\begin{aligned} {W}_{(A)} = W_a({\varvec{U}}) =W^*({\varvec{U}}^*,J) \, . \end{aligned}$$

The spectral representation

6 $$\begin{aligned} {\varvec{U}}^* = \sum _{i=1}^3 \lambda ^*_i {\varvec{u}}_i\otimes {\varvec{u}}_i \, , \end{aligned}$$

where $\lambda ^*_i = J^{-\frac{1}{3}}\lambda _i$, $\otimes$ denotes the dyadic product and, $\lambda _i$ and ${\varvec{u}}_i$ are an eigenvalue and an orthonormal eigenvector of ${\varvec{U}}$, respectively. In view of (6),

7 $$\begin{aligned} {W}_{(A)} = W^*({\varvec{U}}^*, J) = W(\lambda ^*_1,\lambda ^*_2,\lambda ^*_3,{\varvec{u}}_1,{\varvec{u}}_2,{\varvec{u}}_2,J) \, . \end{aligned}$$

Since $\lambda ^*_1\lambda ^*_2\lambda ^*_3=1$ only 6 of the 7 arguments in (7) are independent. The strain energy function *W* must satisfy the *P*-property described in^13^, that is associated with the symmetrical property of *W* and the unique value of *W* when two or more of the principal axes has the same value.

### Stress

The Cauchy stress for a compressible solid is

8 $$\begin{aligned} {\varvec{T}}= {\displaystyle \frac{2}{J}}{\varvec{F}}{\displaystyle \frac{\partial {W}_{(A)}}{\partial {\varvec{C}}}}{\varvec{F}}^T \, \end{aligned}$$

and for an incompressible solid

9 $$\begin{aligned} {\varvec{T}}= 2{\varvec{F}}{\displaystyle \frac{\partial {W}_{(A)}}{\partial {\varvec{C}}}}{\varvec{F}}^T - p{\varvec{I}}\, , \end{aligned}$$

where *p* is the Lagrange multiplier associated with the constraint $\det {\varvec{F}}= 1$ and ${\varvec{I}}$ is the identity tensor. Following the work of^10,17^, the Cauchy stress ${\varvec{T}}$ with respect to the Eulerian orthonormal basis $\{ {\varvec{v}}_1,{\varvec{v}}_2,{\varvec{v}}_3 \}$, where ${\varvec{v}}_i = {\varvec{R}}{\varvec{u}}_i$ and ${\varvec{R}}= {\varvec{F}}{\varvec{U}}^{-1}$ takes the form

10 $$\begin{aligned} {\varvec{T}}= \sum _{i,j=1}^3 t_{ij} {\varvec{v}}_i\otimes {\varvec{v}}_j \, , {\quad }t_{ij} = {\varvec{v}}_i\cdot {\varvec{T}}{\varvec{v}}_j \, , \end{aligned}$$

where

11 $$\begin{aligned} J t_{ii}= & {} \lambda ^*_i {\displaystyle \frac{\partial W}{\partial \lambda ^*_i}} - p^* \, , {\quad }J t_{ij} = {\displaystyle \frac{\lambda ^*_i\lambda ^*_j}{\lambda ^{*2}_i - \lambda ^{*2}_j}} \left( {\displaystyle \frac{\partial W}{\partial {\varvec{u}}_i}}\cdot {\varvec{u}}_j - {\displaystyle \frac{\partial W}{\partial {\varvec{u}}_j}}\cdot {\varvec{u}}_i \right) \, , {\quad }i\ne j \, ,\end{aligned}$$

12 $$\begin{aligned} p^*= & {} {\displaystyle \frac{1}{3}}\sum _{i=1}^3 \lambda ^*_i {\displaystyle \frac{\partial W}{\partial \lambda ^*_i}} - J {\displaystyle \frac{\partial W}{\partial J}} \, , \end{aligned}$$

for compressible elastic solids and in the case for an incompressible solid ($J=1$), we have,

13 $$\begin{aligned} t_{ii} = \lambda _i {\displaystyle \frac{\partial W}{\partial \lambda _i}} - p \, , {\quad }t_{ij} = {\displaystyle \frac{\lambda _i\lambda _j}{\lambda ^2_i - \lambda ^2_j}} \left( {\displaystyle \frac{\partial W}{\partial {\varvec{u}}_i}}\cdot {\varvec{u}}_j - {\displaystyle \frac{\partial W}{\partial {\varvec{u}}_j}}\cdot {\varvec{u}}_i \right) \, , {\quad }i\ne j \, . \end{aligned}$$

The nominal stress

14 $$\begin{aligned} {\varvec{S}}= J{\varvec{F}}^{-1}{\varvec{T}}= \sum _{i,j=1}^3 {\displaystyle \frac{Jt_{ij}}{\lambda _i}} {\varvec{u}}_i\otimes {\varvec{v}}_j \, . \end{aligned}$$

It is clear from the above that hydrostatic stress for a compressible material

15 $$\begin{aligned} {\displaystyle \frac{\text{ tr }{\varvec{T}}}{3}} = {\displaystyle \frac{1}{3}}\sum _{i=1}^3 t_{ii} = {\displaystyle \frac{\partial W}{\partial J}}(\lambda ^*_1,\lambda ^*_2,\lambda ^*_3,{\varvec{u}}_1,{\varvec{u}}_2,{\varvec{u}}_2,J) \, . \end{aligned}$$

In the incompressibility limit, the value of ${\displaystyle \lim _{J\rightarrow 1} {\displaystyle \frac{\partial W}{\partial J}} }$ and the appropriate properties of *W* are discussed in Shariff and Parker^38^. We note that all the proposed strain energy functions in this paper are consistent with the strain energy functions proposed by Shariff and Parker^38^. The deformation dependent bulk modulus is defined as^39^

16 $$\begin{aligned} B({\varvec{U}}) = {\displaystyle \frac{\partial ^2W}{\partial J^2}}(\lambda ^*_1,\lambda ^*_2,\lambda ^*_3,{\varvec{u}}_1,{\varvec{u}}_2,{\varvec{u}}_2,J) \, . \end{aligned}$$

The ground-state bulk modulus is defined as

17 $$\begin{aligned} \chi = B({\varvec{I}}) \, . \end{aligned}$$

## Generalized strain

Consider a set of general class of Lagrangean strain tensor ${{\varvec{F}}}_{(\alpha )}$, similar to that defined by Hill^1^,

18 $$\begin{aligned} {{\varvec{F}}}_{(\alpha )}({\varvec{U}}) = \sum _{i=1}^3 f_\alpha (\lambda ^*_i) {\varvec{u}}_i\otimes {\varvec{u}}_i \, , {\quad }\alpha \in N \end{aligned}$$

where $N =\{ 1,2,3, \ldots \}$ is the set of natural numbers excluding 0 and $f_\alpha :(0,\infty )\rightarrow {\mathbb {R}}$ is a monotonic increasing function, i.e, $f'_\alpha (\lambda ^*_i) > 0$, such that

19 $$\begin{aligned} f_\alpha (1)=0 \, , {\quad }f_\alpha '(1) = 1 \, . \end{aligned}$$

We could also include, when appropriate, $f_\alpha$ to represent physical strain measures with the extreme deformation values

20 $$\begin{aligned} f_\alpha (\lambda ^*_i \rightarrow \infty ) =\infty \, , {\quad }f_\alpha (\lambda ^*_i \rightarrow 0) = -\infty \, . \end{aligned}$$

An example of a strain measure commonly used in the literature that satisfies the above properties is

21 $$\begin{aligned} \ln ({\varvec{U}}^*) = \sum _{i=1}^3 \ln (\lambda ^*_i) {\varvec{u}}_i\otimes {\varvec{u}}_i \, , {\quad }f_\alpha (x) = \ln x \, . \end{aligned}$$

A strain function that could be of interest, which is similar to the Ogden’s strain function^2^, is

22 $$\begin{aligned} f_\alpha (x) = {\displaystyle \frac{1}{m}} \sum _{n=1}^m {\displaystyle \frac{x^{{\bar{\alpha }}_n} -1}{{\bar{\alpha }}_n}} \, \, , {\quad }x > 0 \, , \end{aligned}$$

where ${\bar{\alpha }}$ is a real number. We strongly emphasize that we are not concerned with proposing prototypes of the strain function $f_\alpha$, such as those expressed in (21) and (22). An objective of this paper is to construct constitutive equations that depend explicitly on the arbitrary functions $f_\alpha$ and $g_\alpha$ (defined below), and are consistent with infinitesimal elasticity.

We define a volumetric strain

23 $$\begin{aligned} g_\alpha (J) \, , {\quad }\alpha \in N \, , \end{aligned}$$

where $g_\alpha :(0,\infty )\rightarrow {\mathbb {R}}$ is a monotonic increasing function such that

24 $$\begin{aligned} g_\alpha (1) = 0 \, , {\quad }g_\alpha '(1) = 1\, . \end{aligned}$$

We also include, if required, $g_\alpha$ to represent physical volumetric strain measures with the extreme deformation values

25 $$\begin{aligned} g_\alpha (J \rightarrow \infty ) =\infty \, , {\quad }g_\alpha (J\rightarrow 0) = -\infty \, . \end{aligned}$$

An example of a volumetric strain is

26 $$\begin{aligned} g_1 (J) = \ln J \, . \end{aligned}$$

Note that in view of (18), we have, for example

27 $$\begin{aligned} \text{ tr }( {{\varvec{F}}}_{(\alpha )} {{\varvec{F}}}_{(\beta )} ) = \sum _{i=1}^3 f_\alpha (\lambda _i^*)f_\beta (\lambda _i^*) \, , {\quad }\alpha , \beta \in N \, . \end{aligned}$$

It is clear from the properties of $f_\alpha$ that

28 $$\begin{aligned} f_\alpha (\lambda _i^*)f_\beta (\lambda _i^*) \ge 0 \, . \end{aligned}$$

### Infinitesimal strain

In infinitesimal strain

29 $$\begin{aligned} \mid {\varvec{F}}-{\varvec{I}}\mid = \mid {\displaystyle \frac{\partial {\varvec{u}}}{\partial {\varvec{x}}}} \mid = O(e) \, , \end{aligned}$$

where ${\varvec{u}}$ is the displacement vector, $\mid \bullet \mid$ is an appropriate norm and the magnitude of *e* is much less than unity. Up to *O*(*e*), we have

30 $$\begin{aligned} {\varvec{U}}-{\varvec{I}}\approx {\varvec{E}}\, , {\quad }{\varvec{U}}^* -{\varvec{I}}\approx {\varvec{E}}^* \, , {\quad }f_\alpha (\lambda _i) \approx \lambda _i-1 = e_i \, , {\quad }f_\alpha (\lambda ^*_i) \approx \lambda ^*_i -1 =e^*_i \, , \end{aligned}$$

where $e_i$ is an eigenvalue of the infinitesimal strain ${\varvec{E}}$ (we do not distinguish the eigenvectors of ${\varvec{U}}$ and ${\varvec{E}}$) and $e^*_i$ is an eigenvalue of

31 $$\begin{aligned} {\varvec{E}}^* = {\varvec{E}}- {\displaystyle \frac{1}{3}}(\text{ tr }{\varvec{E}}) {\varvec{I}}\, . \end{aligned}$$

Spectrally, we can express

32 $$\begin{aligned} {\varvec{E}}= \sum _{i=1}^3 e_i {\varvec{u}}_i\otimes {\varvec{u}}_i \, , {\quad }{\varvec{E}}^* = \sum _{i=1}^3 e^*_i {\varvec{u}}_i\otimes {\varvec{u}}_i \, , \end{aligned}$$

where the eigenvalues

33 $$\begin{aligned} e^*_i = e_i - {\displaystyle \frac{e_1+e_2+e_3}{3}} \, . \end{aligned}$$

Up to order *O*(*e*), the volumetric strain

34 $$\begin{aligned} g_\alpha (J) \approx J-1 = \text{ tr }{\varvec{E}}\, . \end{aligned}$$

### Remark

In this paper, the construction of a strain energy function for finite strain deformations that is consistent with infinitesimal elasticity is facilitated via infinitesimal strain elasticity. Hence, in sections “Isotropic” to “General anisotropy”, we start the construction of a finite strain constitutive equation with the development of its infinitesimal strain energy function counterpart.

For the sake of generality, the general constitutive equations given below contain numerous functions of $f_\alpha$ and $g_\alpha$, which may seem unappealing. However, in many occasions only a few $f_\alpha$ and $g_\alpha$ functions are required to model anisotropic solids (see “Example of specific forms of *fα* and *gα* used in experimental fitting” section).

## Isotropic

Let ${W}_{(I)}$ represents the strain energy for an isotropic elastic solid. We then have

35 $$\begin{aligned} {W}_{(I)} = W_I({\varvec{U}}) = W_I({\varvec{Q}}{\varvec{U}}{\varvec{Q}}^T) \, , \end{aligned}$$

where ${\varvec{Q}}$ is an arbitrary rotational tensor and (35) implies that the strain energy ${W}_{(I)}$ can be symmetrically express in terms the principal stretches (spectral invariants) $\lambda _i$.

### Infinitesimal strain

The strain energy function for infinitesimal strain deformations is

36 $$\begin{aligned} {W}_{(I)} = \mu \text{ tr }{\varvec{E}}^2 + {\displaystyle \frac{\lambda }{2}} (\text{ tr }{\varvec{E}})^2 = \mu \text{ tr }{\varvec{E}}^{*2} + {\displaystyle \frac{\chi }{2}} (\text{ tr }{\varvec{E}})^2 \, , \end{aligned}$$

where $\mu$ and $\chi$ are, respectively, the ground state shear and bulk moduli and $\lambda$ is the Lame’s constant. For the purpose of this paper, we express

37 $$\begin{aligned} {W}_{(I)} = \mu \sum _{i=1} e^{*2}_i + {\displaystyle \frac{\chi }{2}} h^2 \, , \end{aligned}$$

where $h =\text{ tr }{\varvec{E}}$. In the case of an incompressible solid, (37) is reduced to

38 $$\begin{aligned} {W}_{(I)} = \mu \sum _{i=1} e^{2}_i \, . \end{aligned}$$

### Finite strain

A finite strain energy function that is consistent with its infinitesimal counterpart (37) is proposed, i.e.,

39 $$\begin{aligned} {W}_{(I)} = \mu \text{ tr }( {{\varvec{F}}}_{(1)} {{\varvec{F}}}_{(2)})+ {\displaystyle \frac{\chi }{2}} g_1(J)g_2(J) + {\phi }_{(I)} = \mu \sum _{i=1}^3 f_1(\lambda ^*_i)f_2(\lambda ^*_i) + {\displaystyle \frac{\chi }{2}} g_1(J)g_2(J) + {\phi }_{(I)} \, , \end{aligned}$$

where the “higher order” term ${\phi }_{(I)}$ (which depends on $\lambda _i$) satisfies the *P*-property and the conditions

40 $$\begin{aligned} {\phi }_{(I)} = 0 \, , {\quad }{\displaystyle \frac{\partial {\phi }_{(I)}}{\partial {\varvec{U}}}} = {\varvec{0}}\, , {\quad }{\displaystyle \frac{\partial ^2 {\phi }_{(I)}}{\partial {\varvec{U}}\partial {\varvec{U}}}}= {\varvec{0}}\, \end{aligned}$$

at ${\varvec{F}}={\varvec{I}}$. We note that, in view of (40), the function ${\phi }_{(I)}$ does not contribute to the infinitesimal strain energy function. In the case of an incompressible material, we propose

41 $$\begin{aligned} {W}_{(I)} = \mu \sum _{i=1}^3 f_1(\lambda _i)f_2(\lambda _i) + {\phi }_{(I)} \, . \end{aligned}$$

For neatness, we have used the same expression for ${\phi }_{(I)}$ in (39) and (41) although they are, generally, different functions.

The weighted Cauchy stress takes the form

42 $$\begin{aligned} J{\varvec{T}}= \sum _{i=1}^3 \left( \lambda ^*_i {\displaystyle \frac{\partial {W}_{(I)}}{\partial \lambda ^*_i}} - p^* \right) {\varvec{v}}_i\otimes {\varvec{v}}_i \, . \end{aligned}$$

Examples of strain energy functions in the literature that can be written in the forms (39) and (41) are given below:

(a) For compressible materials with ${\Phi }_{(I)}=0$:

1. The Hencky^40^ strain energy function
  43 $$\begin{aligned} {W}_{(I)} = \mu \sum _{i=1}^3 (\ln (\lambda ^*_i))^2 + {\displaystyle \frac{\chi }{2}} (\ln J)^2 = \mu \sum _{i=1}^3 (\ln (\lambda _i))^2 + {\displaystyle \frac{\lambda }{2}} (\ln J)^2 \, .\end{aligned}$$
  In this case, we have,
  44 $$\begin{aligned} f_1(\lambda ^*_i)=f_2(\lambda ^*_i) = \ln (\lambda ^*_i) \, , {\quad }g_1(J)=g_2(J) = \ln J \, . \end{aligned}$$
2. A Mooney-Rivlin strain energy function
  45 $$\begin{aligned} {W}_{(I)}= & {} c_1 \left( \sum _{i=1}^3 \lambda ^{*2}_i - 3\right) + c_2\left( \sum _{i=1}^3 {\displaystyle \frac{1}{\lambda ^{*2}_i}} - 3\right) + {\displaystyle \frac{\chi }{2}}(J-1)^2 \nonumber \\= & {} c_1 \sum _{i=1}^3( \lambda ^{*2}_i - 2\ln \lambda ^*_i -1) + c_2\sum _{i=1}^3( {\displaystyle \frac{1}{\lambda ^{*2}_i}} + 2\ln \lambda ^*_i -1) + {\displaystyle \frac{\chi }{2}}(J-1)^2 \, . \end{aligned}$$
  In this case, we have,
  46 $$\begin{aligned} \mu= & {} 2(c_1+c_2) \, , {\quad }f_1(\lambda ^*_i)=f_2(\lambda ^*_i)=f(\lambda ^*_i) \, , \nonumber \\ f(\lambda ^*_i)^2= & {} {\displaystyle \frac{1}{\mu }} \left( c_1( \lambda ^{*2}_i - 2\ln \lambda ^*_i -1) + c_2\left( {\displaystyle \frac{1}{\lambda ^{*2}_i}} + 2\ln \lambda ^*_i -1\right) \right) \ge 0 \, ,\nonumber \\ g_1(J)= & {} g_2(J) = (J-1) \, , \end{aligned}$$
  for appropriate values of $c_1$ and $c_2$. Note that, we can also compare our model with Mooney-Rivlin strain energy function, where $f_1\ne f_2$. For example,
  47 $$\begin{aligned} f_1(\lambda ^*_i)= & {} (\lambda ^*_i-1) \, ,\nonumber \\ f_2(\lambda ^*_i)= & {} {\displaystyle \frac{1}{\mu (\lambda ^*_i-1)}} \left( c_1( \lambda ^{*2}_i - 2\ln \lambda ^*_i -1) + c_2\left( {\displaystyle \frac{1}{\lambda ^{*2}_i}} + 2\ln \lambda ^*_i -1\right) \right) \, , \end{aligned}$$
  taking note that
  48 $$\begin{aligned} \lim _{\lambda ^*_i \rightarrow 1} f_2(\lambda ^*_i) = 0 \, , {\quad }\lim _{\lambda ^*_i \rightarrow 1} f'_2(\lambda ^*_i) = 1 \, . \end{aligned}$$

(b) Incompressible materials with ${\Phi }_{(I)}=0$:

1. The Valanis and Landel^36^ form
  49 $$\begin{aligned} {W}_{(I)} = \sum _{i=1}^3 r(\lambda _i) \, . \end{aligned}$$
  In this case
  50 $$\begin{aligned} f_1(\lambda _i)=f_2(\lambda _i) =f(\lambda _i) \, , {\quad }f(\lambda _i)^2 = {\displaystyle \frac{1}{\mu }} r(\lambda _i) \, \, , \end{aligned}$$
  with the conditions
  51 $$\begin{aligned} r(1)=r'(1)=0 \, , {\quad }r''(1) = 2\mu \, . \end{aligned}$$
2. Ogden^2^ strain energy function
  52 $$\begin{aligned} {W}_{(I)} = \sum _{r} {\displaystyle \frac{\mu _r}{\alpha _r}} (\lambda _1^{\alpha _r} + \lambda _2^{\alpha _r} + \lambda _3^{\alpha _r} - 3) \, . \end{aligned}$$
  In this case, we have,
  53 $$\begin{aligned} \mu= & {} {\displaystyle \frac{1}{2}}\sum _{r} \mu _r\alpha _r \, , {\quad }f_1(\lambda _i)=f_2(\lambda _i) =f(\lambda _i) \, , \nonumber \\ f(\lambda _i)^2= & {} {\displaystyle \frac{1}{\mu }} \sum _{r} {\displaystyle \frac{\mu _r}{\alpha _r}} (\lambda _i^{\alpha _r} - \alpha _r\ln (\lambda _i) - 1) \ge 0 \, , \end{aligned}$$
  for appropriate values of the material constants $\mu _r$ and $\alpha _r$. It is worth noting that
  54 $$\begin{aligned} \lambda _i^{\alpha _r} - \alpha _r\ln (\lambda _i) - 1 \ge 0 \, . \end{aligned}$$

Remark: In sections “Transversely Isotropic with a unit preferred direction *a*” and “Two preferred direction elastic solid” below, we discuss elastic solids with one and two preferred directions. In some of these solids, the mechanical influence of compressed fibres is negligible or is different from stretched fibres and in some soft tissue solids, the influence of fibre dispersion could be relevant in modelling constitutive equations: In Appendices A and B (Supplementary information), we illustrate how the strain energy functions developed in sections “Transversely Isotropic with a unit preferred direction *a*” and “Two preferred direction elastic solid” can be easily amended to take account of these influences.

## Transversely Isotropic with a unit preferred direction ${\varvec{a}}$

Let ${W}_{(T)}$ represents the strain energy for a transversely isotropic elastic solid. We then have

55 $$\begin{aligned} {W}_{(T)} = {\bar{W}}_T({\varvec{U}},{\varvec{a}}\otimes {\varvec{a}}) = W_T({\varvec{U}},{\varvec{a}}) = W_T({\varvec{Q}}{\varvec{U}}{\varvec{Q}}^T,{\varvec{Q}}{\varvec{a}}) \, . \end{aligned}$$

Following the work of Shariff^14^, we can express the strain energy function in terms of the spectral invariants

56 $$\begin{aligned} \lambda _i \, , {\quad }a_i = {\varvec{u}}_i\cdot {\varvec{a}}= {\varvec{Q}}{\varvec{u}}_i\cdot {\varvec{Q}}{\varvec{a}}\, . \end{aligned}$$

Since, ${\varvec{a}}$ is a unit vector, we have,

57 $$\begin{aligned} \sum _{i=1}^3 \zeta _i = 1 \, , {\quad }\zeta _i=a^2_i \, \end{aligned}$$

and hence only 5 of the 6 invariants in (56) are independent^31,32^.

### Infinitesimal strain

The infinitesimal strain energy function is^41^

58 $$\begin{aligned} {W}_{(T)} = {\bar{a}}_1 \text{ tr }{\varvec{E}}^2 + {\bar{a}}_2 (\text{ tr }{\varvec{E}})^2 + {\bar{a}}_3 \text{ tr }({\varvec{A}}{\varvec{E}}^2) + {\bar{a}}_4 (\text{ tr }({\varvec{A}}{\varvec{E}}))^2 + {\bar{a}}_5 \text{ tr }({\varvec{A}}{\varvec{E}})\text{ tr }{\varvec{E}}\, , \end{aligned}$$

where ${\varvec{A}}= {\varvec{a}}\otimes {\varvec{a}}$. The material constants ${\bar{a}}_i$ ($i=1,2,\ldots , 5$) can be be described in terms of physical parameters as shown below:

59 $$\begin{aligned} {\bar{a}}_1= & {} {\displaystyle \frac{a_{11}-a_{12}}{2}} \, , {\quad }{\bar{a}}_2 = {\displaystyle \frac{a_{12}}{2}} \, , {\quad }{\bar{a}}_3 = a_{44}-a_{11}+a_{12} \, , {\quad }{\bar{a}}_4 = {\displaystyle \frac{a_{33}}{2}} + {\displaystyle \frac{a_{11}}{2}} - a_{44} - a_{13} \, , \nonumber \\ {\bar{a}}_5= & {} a_{13}-a_{12} \, , \end{aligned}$$

where

60 $$\begin{aligned} a_{11}= & {} {\displaystyle \frac{1-\nu _a\nu _{zp}}{E_pE_a D}} \, , {\quad }a_{12} = {\displaystyle \frac{\nu _p + \nu _{zp}\nu _a}{E_pE_a D}} \, , {\quad }a_{13} = {\displaystyle \frac{\nu _a(1+\nu _p)}{E_p^2 D}} \, , {\quad }a_{33}={\displaystyle \frac{1-\nu _p^2}{E_p^2 D}} \, , {\quad }a_{44} = 2 \mu _a \, , \end{aligned}$$

61 $$\begin{aligned} D= & {} {\displaystyle \frac{(1+\nu _p)(1-\nu _p - 2\nu _a\nu _{zp})}{E_p^2E_a}} \, . \end{aligned}$$

Here $\nu _p$ is the Poisson ratio in a particular direction on the plane of symmetry, when the material is extended in a direction on the plane of symmetry perpendicular to the particular direction, $\nu _a$ is the Poisson ratio in the preferred direction when the material is extended in the plane of symmetry, $E_p$ is the Young’s Modulus in the plane of symmetry normal to the preferred direction ${\varvec{a}}$, $\mu _a$ is the shear modulus in the preferred direction and $E_a$ is the Young’s modulus in the preferred direction. Take note that we have also the relation

62 $$\begin{aligned} {\displaystyle \frac{\nu _a}{E_p}} = {\displaystyle \frac{\nu _{zp}}{E_a}}\, , \end{aligned}$$

where $\nu _{zp}$ is the Poisson ratio in any direction on the plane of symmetry, when the material is extended in the preferred direction.

We can express (58) in the form

63 $$\begin{aligned} {W}_{(T)} = W_T ({\varvec{E}}^*,h) = {\bar{a}}_1 \text{ tr }{\varvec{E}}^{*2} + {a}_{(2)} h^2 + {\bar{a}}_3 {\varvec{a}}\cdot {\varvec{E}}^{*2}{\varvec{a}}+ {\bar{a}}_4 ({\varvec{a}}\cdot {\varvec{E}}^*{\varvec{a}})^2 + {a}_{(5)} h {\varvec{a}}\cdot {\varvec{E}}^*{\varvec{a}}\, , \end{aligned}$$

where

64 $$\begin{aligned} {a}_{(2)} = {\displaystyle \frac{{\bar{a}}_1}{3}} + {\bar{a}}_2 + {\displaystyle \frac{{\bar{a}}_3}{9}} + {\displaystyle \frac{{\bar{a}}_4}{9}} + {\displaystyle \frac{{\bar{a}}_5}{3}} \, , {\quad } {a}_{(5)} = {\displaystyle \frac{2{\bar{a}}_3}{3}} + {\displaystyle \frac{2{\bar{a}}_4}{3}} + {\bar{a}}_5 \, . \end{aligned}$$

The infinitesimal hydrostatic stress

65 $$\begin{aligned} {\displaystyle \frac{ \text{ tr }{\varvec{T}}}{3}} = {\displaystyle \frac{\partial W_T}{\partial h}}({\varvec{E}}^*,h) = 2 {a}_{(2)} h + {a}_{(5)} {\varvec{a}}\cdot {\varvec{E}}^*{\varvec{a}}\, . \end{aligned}$$

The ground-state bulk modulus then takes the form

66 $$\begin{aligned} \chi = {\displaystyle \frac{\partial ^2 W_T}{\partial h^2}}({\varvec{E}}^*,h) = 2 {a}_{(2)} \, . \end{aligned}$$

It can be easily shown that, in the incompressible limit, as ${\displaystyle \nu _{zp} \rightarrow 0.5 }$ and ${\displaystyle 1-\nu _a-\nu _p \rightarrow 0}$^42^ the ground-state bulk modulus ${\displaystyle \chi \rightarrow \infty }$. It is clear from (65) that, since,

67 $$\begin{aligned} \lim _{\nu _{zp} \rightarrow 0.5 , 1-\nu _a-\nu _p \rightarrow 0} {a}_{(5)} \end{aligned}$$

exists, we have

68 $$\begin{aligned} h = \text{ tr }{\varvec{E}}\rightarrow 0 {\quad }\text{ as } {\quad }\chi \rightarrow \infty \, . \end{aligned}$$

In spectral form,

69 $$\begin{aligned} \text{ tr }{\varvec{E}}^{*2} = \sum _{i=1}^3 e^{*2}_i \, , {\quad }{\varvec{a}}\cdot {\varvec{E}}^*{\varvec{a}}= \sum _{i=1}^3 \zeta _i e^*_i \, , {\quad }{\varvec{a}}\cdot {\varvec{E}}^{*2} {\varvec{a}}= \sum _{i=1}^3 \zeta _i e^{*2}_i \, . \end{aligned}$$

### Finite strain

Using (63) and (69), we easily construct a finite strain energy that it is consistent with its infinitesimal counterpart, i.e.

70 $$\begin{aligned} {W}_{(T)}= & {} {\bar{a}}_1 \text{ tr }( {{\varvec{F}}}_{(1)} {{\varvec{F}}}_{(2)}) + {a}_{(2)}g_1(J)g_2(J) + {\bar{a}}_3 {\varvec{a}}\cdot {{\varvec{F}}}_{(3)} {{\varvec{F}}}_{(4)} {\varvec{a}}\nonumber \\&+ {\bar{a}}_4({\varvec{a}}\cdot {{\varvec{F}}}_{(5)}{\varvec{a}})({\varvec{a}}\cdot {{\varvec{F}}}_{(6)}{\varvec{a}}) + {a}_{(5)} g_3(J){\varvec{a}}\cdot {{\varvec{F}}}_{(7)}{\varvec{a}}+ {\phi }_{(I)} \, \end{aligned}$$

or, alternatively,

71 $$\begin{aligned} {W}_{(T)}= & {} {\bar{a}}_1 \sum _{i=1}^3 f_1(\lambda ^*_i)f_2(\lambda ^*_i) + {a}_{(2)} g_1(J)g_2(J) + {\bar{a}}_3 \sum _{i=1}^3 \zeta _i f_3(\lambda ^*_i)f_4(\lambda ^*_i) \nonumber \\&+{\bar{a}}_4 \left( \sum _{i=1}^3 \zeta _if_5(\lambda ^*_i)\right) \left( \sum _{i=1}^3 \zeta _if_6(\lambda ^*_i)\right) + {a}_{(5)} g_3(J) \sum _{i=1}^3 \zeta _if_7(\lambda ^*_i) + {\phi }_{(I)} \, , \end{aligned}$$

where the higher order function ${\phi }_{(I)}$ (for convenient, we use the same expression for all anisotropic material discussed in this paper, although they are, generally, different functions.) has the properties given in “Finite strain” section and ${\phi }_{(I)}$ depends on the spectral invariants $\lambda _i$ and $a_i$.

The weighted Cauchy stress takes the form

72 $$\begin{aligned} J{\varvec{T}}= \sum _{i=1}^3 \left( \lambda ^*_i {\displaystyle \frac{\partial {W}_{(T)}}{\partial \lambda ^*_i}} - p^*\right) {\varvec{v}}_i\otimes {\varvec{v}}_i + \sum _{i\ne j}^3 {\displaystyle \frac{2a_ia_j \lambda ^*_i\lambda ^*_j}{\lambda ^{*2}_i - \lambda ^{*2}_j}}\left( {\displaystyle \frac{\partial {W}_{(T)}}{\partial \zeta _i}} - {\displaystyle \frac{\partial {W}_{(T)}}{\partial \zeta _j}} \right) {\varvec{v}}_i\otimes {\varvec{v}}_j \, . \end{aligned}$$

## Two preferred direction elastic solid

Consider an elastic material with preferred unit directions ${\varvec{a}}$ and ${\varvec{b}}$, where the unit vectors ${\varvec{a}}$ and ${\varvec{b}}$ are independent. The strain energy

73 $$\begin{aligned} {W}_{(P)} = {\bar{W}}_P({\varvec{U}},{\varvec{a}}\otimes {\varvec{a}},{\varvec{b}}\otimes {\varvec{b}}) = W_P({\varvec{U}},{\varvec{a}},{\varvec{b}}) = W_P({\varvec{Q}}{\varvec{U}}{\varvec{Q}}^T,{\varvec{Q}}{\varvec{a}},{\varvec{Q}}{\varvec{b}}) \, . \end{aligned}$$

Hence, we can express ${W}_{(P)}$ in terms of the spectral invariants^17,30^

74 $$\begin{aligned} \lambda _i \, , {\quad }a_i \, , {\quad }\iota _i = {\varvec{u}}_i\cdot {\varvec{b}}={\varvec{Q}}{\varvec{u}}_i\cdot {\varvec{Q}}{\varvec{b}}\, . \end{aligned}$$

We note that in view of (57) and the relations

75 $$\begin{aligned} \sum _{i=1} \gamma _i = 1 \, , {\quad }\gamma _i=\iota ^2_i \, , \end{aligned}$$

only 7 of the 9 invariants in (74) are independent and they formed the minimal/irreducible integrity basis^30,32^.

### Infinitesimal strain

Modifying the work of Shariff and Bustamante^30^, we have the strain energy

76 $$\begin{aligned} {W}_{(P)}= & {} W_p({\varvec{E}}^*,h)=b_1\text{ tr }{\varvec{E}}^{*2} + b_2{\varvec{a}}\cdot {\varvec{E}}^{*2}{\varvec{a}}+ b_3 ({\varvec{a}}\cdot {\varvec{E}}^*{\varvec{a}})^2 + b_4{\varvec{b}}\cdot {\varvec{E}}^{*2}{\varvec{b}}+ b_5({\varvec{b}}\cdot {\varvec{E}}^*{\varvec{b}})^2 \nonumber \\&+ b_6({\varvec{a}}\cdot {\varvec{b}}){\varvec{a}}\cdot {\varvec{E}}^{*2}{\varvec{b}}+ b_7[({\varvec{a}}\cdot {\varvec{b}}){\varvec{a}}\cdot {\varvec{E}}^*{\varvec{b}}]^2 + b_8 ({\varvec{a}}\cdot {\varvec{E}}^*{\varvec{a}})({\varvec{b}}\cdot {\varvec{E}}^*{\varvec{b}}) + b_9({\varvec{a}}\cdot {\varvec{b}})({\varvec{a}}\cdot {\varvec{E}}^*{\varvec{b}})({\varvec{a}}\cdot {\varvec{E}}^*{\varvec{a}}) \nonumber \\&+b_{10}({\varvec{a}}\cdot {\varvec{b}})({\varvec{a}}\cdot {\varvec{E}}^*{\varvec{b}})({\varvec{b}}\cdot {\varvec{E}}^*{\varvec{b}}) + b_{11}h({\varvec{a}}\cdot {\varvec{E}}^*{\varvec{a}}) + b_{12}h({\varvec{b}}\cdot {\varvec{E}}^*{\varvec{b}}) + b_{13}h({\varvec{a}}\cdot {\varvec{b}})({\varvec{a}}\cdot {\varvec{E}}^*{\varvec{b}}) + {\displaystyle \frac{\chi }{2}}h^2 \, . \end{aligned}$$

The mean hydrostatic stress is

77 $$\begin{aligned} {\displaystyle \frac{ \text{ tr }{\varvec{T}}}{3}} = {\displaystyle \frac{\partial W_p}{\partial h}}({\varvec{E}}^*,h) = b_{11}({\varvec{a}}\cdot {\varvec{E}}^*{\varvec{a}}) + b_{12}({\varvec{b}}\cdot {\varvec{E}}^*{\varvec{b}}) + b_{13}({\varvec{a}}\cdot {\varvec{b}})({\varvec{a}}\cdot {\varvec{E}}^*{\varvec{b}}) + h\chi \, . \end{aligned}$$

The bulk modulus

78 $$\begin{aligned} \chi = {\displaystyle \frac{\partial ^2W_p}{\partial h^2}}({\varvec{E}}^*,h) \, . \end{aligned}$$

#### Mechanically equivalent material

For a mechanically equivalent material we simply let

79 $$\begin{aligned} b_2=b_4 \, , {\quad }b_3=b_5 \, , {\quad }b_9=b_{10} \, , {\quad }b_{11}=b_{12} \, , \end{aligned}$$

and we have only 10 material constants [In Shariff and Bustamante^30^, they mistakenly evaluate 11 material constants.] to characterize its mechanical behaviour. Hence, we have, the strain energy

80 $$\begin{aligned} {W}_{(M)}=W_M({\varvec{E}}^*,h)= & {} c_1\text{ tr }{\varvec{E}}^{*2} + c_2[{\varvec{a}}\cdot {\varvec{E}}^{*2}{\varvec{a}}+{\varvec{b}}\cdot {\varvec{E}}^{*2}{\varvec{b}}]+ c_3 [({\varvec{a}}\cdot {\varvec{E}}^*{\varvec{a}})^2 + ({\varvec{b}}\cdot {\varvec{E}}^*{\varvec{b}})^2] \nonumber \\&+ c_4({\varvec{a}}\cdot {\varvec{b}}){\varvec{a}}\cdot {\varvec{E}}^{*2}{\varvec{b}}+ c_5[({\varvec{a}}\cdot {\varvec{b}}){\varvec{a}}\cdot {\varvec{E}}^*{\varvec{b}}]^2 + c_6({\varvec{a}}\cdot {\varvec{E}}^*{\varvec{a}})({\varvec{b}}\cdot {\varvec{E}}^*{\varvec{b}}) \nonumber \\&+ c_7 [ ({\varvec{a}}\cdot {\varvec{b}})({\varvec{a}}\cdot {\varvec{E}}^*{\varvec{b}})({\varvec{a}}\cdot {\varvec{E}}^*{\varvec{a}}) + ({\varvec{a}}\cdot {\varvec{b}})({\varvec{a}}\cdot {\varvec{E}}^*{\varvec{b}})({\varvec{b}}\cdot {\varvec{E}}^*{\varvec{b}})] \nonumber \\&+c_8[h({\varvec{a}}\cdot {\varvec{E}}^*{\varvec{a}}) + h({\varvec{b}}\cdot {\varvec{E}}^*{\varvec{b}})] + c_9h({\varvec{a}}\cdot {\varvec{b}})({\varvec{a}}\cdot {\varvec{E}}^*{\varvec{b}})] + {\displaystyle \frac{\chi }{2}}h^2 \, , \end{aligned}$$

where $c_\alpha$ ($\alpha =1,2,\ldots 9$) are ground state material constants.

#### Orthotropic elastic solid

In the case of an orthotropic material, where the preferred directions ${\varvec{a}}$ and ${\varvec{b}}$ are orthogonal, we have

81 $$\begin{aligned} {W}_{(O)}= & {} W_O({\varvec{E}}^*,h)=d_1\text{ tr }{\varvec{E}}^{*2} + d_2{\varvec{a}}\cdot {\varvec{E}}^{*2}{\varvec{a}}+ d_3 ({\varvec{a}}\cdot {\varvec{E}}^*{\varvec{a}})^2 + d_4{\varvec{b}}\cdot {\varvec{E}}^{*2}{\varvec{b}}+ d_5({\varvec{b}}\cdot {\varvec{E}}^*{\varvec{b}})^2 \nonumber \\&+ d_6 ({\varvec{a}}\cdot {\varvec{E}}^*{\varvec{a}})({\varvec{b}}\cdot {\varvec{E}}^*{\varvec{b}}) + + d_7h({\varvec{a}}\cdot {\varvec{E}}^*{\varvec{a}}) + d_8h({\varvec{b}}\cdot {\varvec{E}}^*{\varvec{b}}) + {\displaystyle \frac{\chi }{2}}h^2 \, , \end{aligned}$$

where $d_1,d_2, \ldots d_8$ are ground state constants.

### Finite strain

Following the work of sections “Isotropic” and “Transversely Isotropic with a unit preferred direction ***a***” sections, we propose the strain energy function

82 $$\begin{aligned} {W}_{(P)}= & {} b_1 \sum _{i=1}^3 f_1(\lambda ^*_i)f_2(\lambda ^*_i) + b_2 \sum _{i=1}^3 \zeta _i f_3(\lambda ^*_i)f_4(\lambda ^*_i)+ b_3\sum _{i=1}^3 \zeta _if_5(\lambda ^*_i)\sum _{i=1}^3 \zeta _if_6(\lambda ^*_i) \nonumber \\&+b_4 \sum _{i=1}^3 \gamma _i f_7(\lambda ^*_i)f_8(\lambda ^*_i)+ b_5 \sum _{i=1}^3 \gamma _if_9(\lambda ^*_i)\sum _{i=1}^3 \gamma _if_{10}(\lambda ^*_i) + b_6\sum _{i=1}^3 \eta _i f_{11}(\lambda ^*_i)f_{12}(\lambda ^*_i) \nonumber \\&+b_7 \sum _{i=1}^3 \eta _i f_{13}(\lambda ^*_i)\sum _{i=1}^3 \eta _i f_{14}(\lambda ^*_i) + b_8\sum _{i=1}^3 \zeta _if_{15}(\lambda ^*_i)\sum _{i=1}^3 \gamma _if_{16}(\lambda ^*_i) \nonumber \\&+ b_9\sum _{i=1}^3 \eta _i f_{17}(\lambda ^*_i)\sum _{i=1}^3 \zeta _if_{18}(\lambda ^*_i) + b_{10}\sum _{i=1}^3 \eta _if_{19}(\lambda ^*_i)\sum _{i=1}^3 \gamma _if_{20}(\lambda ^*_i) \nonumber \\&+ b_{11}g_1(J)\sum _{i=1}^3 \zeta _if_{21}(\lambda ^*_i) + b_{12}g_2(J)\sum _{i=1}^3 \gamma _if_{22}(\lambda ^*_i) \nonumber \\&+ b_{13}g_3(J)\sum _{i=1}^3 \eta _if_{23}(\lambda ^*_i) + {\displaystyle \frac{\chi }{2}}g_4(J)g_5(J) + {\phi }_{(I)} \, , \end{aligned}$$

where $\eta _i=({\varvec{a}}\cdot {\varvec{b}})a_i\iota _i$, ${\phi }_{(I)}$ has the properties given in “Finite strain” section and is a function of $\lambda _i,a_i$ and $\iota _i$.

The weighted Cauchy stress takes the form

83 $$\begin{aligned} J{\varvec{T}}= & {} \sum _{i=1}^3 \left( \lambda ^*_i {\displaystyle \frac{\partial {W}_{(T)}}{\partial \lambda ^*_i}} - p^*\right) {\varvec{v}}_i\otimes {\varvec{v}}_i + \sum _{i\ne j}^3 {\displaystyle \frac{2\lambda ^*_i\lambda ^*_j}{\lambda ^{*2}_i - \lambda ^{*2}_j}}\left\{ \left( {\displaystyle \frac{\partial {W}_{(T)}}{\partial \zeta _i}} - {\displaystyle \frac{\partial {W}_{(T)}}{\partial \zeta _j}}\right) a_ia_j \right. \nonumber \\&+ \left. \left( {\displaystyle \frac{\partial {W}_{(T)}}{\partial \gamma _i}} - {\displaystyle \frac{\partial {W}_{(T)}}{\partial \gamma _j}}\right) \iota _i\iota _j + {\displaystyle \frac{{\varvec{a}}\cdot {\varvec{b}}}{2}}\left( {\displaystyle \frac{\partial {W}_{(T)}}{\partial \eta _i}} - {\displaystyle \frac{\partial {W}_{(T)}}{\partial \eta _j}}\right) (a_i\iota _j + a_j\iota _i) \right\} {\varvec{v}}_i\otimes {\varvec{v}}_j \, . \end{aligned}$$

#### Mechanical equivalent elastic solid

84 $$\begin{aligned} {W}_{(M)}= & {} c_1 \sum _{i=1}^3 f_1(\lambda ^*_i)f_2(\lambda ^*_i) + c_2 \sum _{i=1}^3 (\zeta _i + \gamma _i) f_3(\lambda ^*_i)f_4(\lambda ^*_i) \nonumber \\&+ c_3 \left\{ \sum _{i=1}^3 \zeta _if_5(\lambda ^*_i)\sum _{i=1}^3 \zeta _if_6(\lambda ^*_i)+ \sum _{i=1}^3 \gamma _if_5(\lambda ^*_i)\sum _{i=1}^3 \gamma _if_6(\lambda ^*_i) \right\} + c_4 \sum _{i=1}^3 \eta _i f_7(\lambda ^*_i)f_8(\lambda ^*_i) \nonumber \\&+c_5 \sum _{i=1}^3 \eta _i f_9(\lambda ^*_i)\sum _{i=1}^3 \eta _i f_{10}(\lambda ^*_i) + c_6\sum _{i=1}^3 \zeta _if_{11}(\lambda ^*_i)\sum _{i=1}^3 \gamma _if_{11}(\lambda ^*_i) \nonumber \\&+ c_7\sum _{i=1}^3 \eta _i f_{12}(\lambda ^*_i)\sum _{i=1}^3 (\zeta _i + \gamma _i)f_{13}(\lambda ^*_i)+ c_8 g_1(J)\sum _{i=1}^3 (\zeta _i+\gamma _i) f_{14}(\lambda ^*_i) \nonumber \\&+ c_9g_2(J)\sum _{i=1}^3 \eta _if_{15}(\lambda ^*_i) + {\displaystyle \frac{\chi }{2}}g_4(J)g_5(J) + {\phi }_{(I)} \, . \end{aligned}$$

An orthotropic strain energy function can be easily obtained from (82) by letting $\eta _i=0$.

## General anisotropy

Consider the strain energy ${W}_{(G)}$ that depends of the 4th order classical stiffness tensor ${\mathbb {C}}$, i.e.,

85 $$\begin{aligned} {W}_{(G)} = W_g ({\mathbb {C}},{\varvec{U}}) \, . \end{aligned}$$

Note that $W_g$ must be invariant with respect to the rotation ${\varvec{Q}}$, i.e.

86 $$\begin{aligned} W_g ({\mathbb {C}},{\varvec{U}}) = W_g (\bar{{\mathbb {C}}},{\varvec{Q}}{\varvec{U}}{\varvec{Q}}^T) \, , \end{aligned}$$

where^43^

87 $$\begin{aligned} \bar{{\mathbb {C}}} = \left[ \left( ({\mathbb {C}}:{\varvec{Q}}^T):{\varvec{Q}}^T \right) :{\varvec{Q}}^T \right] \mid {\varvec{Q}}^T \, , \end{aligned}$$

and, the operations  :  and $\mid$ are defined as follows

88 $$\begin{aligned} {\varvec{a}}_1\otimes {\varvec{a}}_2\otimes {\varvec{a}}_3\otimes {\varvec{a}}_4 : {\varvec{b}}_1\otimes {\varvec{b}}_2= & {} ({\varvec{b}}_1\cdot {\varvec{a}}_2) {\varvec{a}}_1\otimes {\varvec{a}}_3\otimes {\varvec{a}}_4\otimes {\varvec{b}}_2 \, , \end{aligned}$$

89 $$\begin{aligned} {\varvec{a}}_1\otimes {\varvec{a}}_2\otimes {\varvec{a}}_3\otimes {\varvec{a}}_4 \mid {\varvec{b}}_1\otimes {\varvec{b}}_2= & {} ({\varvec{b}}_1\cdot {\varvec{a}}_1) {\varvec{b}}_2\otimes {\varvec{a}}_2\otimes {\varvec{a}}_3\otimes {\varvec{a}}_4 \, . \end{aligned}$$

We also have the definition

90 $$\begin{aligned} ({\varvec{a}}_1\otimes {\varvec{a}}_2\otimes {\varvec{a}}_3\otimes {\varvec{a}}_4) ({\varvec{b}}_1\otimes {\varvec{b}}_2) = ({\varvec{a}}_1\otimes {\varvec{a}}_2)({\varvec{a}}_3\cdot {\varvec{b}}_1)({\varvec{a}}_4\cdot {\varvec{b}}_2) \, . \end{aligned}$$

In view of (87),(88) and (89), we obtain

91 $$\begin{aligned} \bar{{\mathbb {C}}} = \sum _{i,j,k,l}c_{ijkl} {\varvec{Q}}{\varvec{u}}_i\otimes {\varvec{Q}}{\varvec{u}}_j\otimes {\varvec{Q}}{\varvec{u}}_k\otimes {\varvec{Q}}{\varvec{u}}_l \, , \end{aligned}$$

where

92 $$\begin{aligned} c_{ijkl} = {\varvec{u}}_i \cdot [{\mathbb {C}}({\varvec{u}}_k\otimes {\varvec{u}}_l)]{\varvec{u}}_j \, . \end{aligned}$$

From (87) and (91), we have

93 $$\begin{aligned} c_{ijkl} = {\varvec{u}}_i \cdot [{\mathbb {C}}({\varvec{u}}_k\otimes {\varvec{u}}_l)]{\varvec{u}}_j = {\varvec{Q}}{\varvec{u}}_i \cdot [\bar{{\mathbb {C}}}({\varvec{Q}}{\varvec{u}}_k\otimes {\varvec{Q}}{\varvec{u}}_l)]{\varvec{Q}}{\varvec{u}}_j \, , \end{aligned}$$

for all rotation ${\varvec{Q}}$ and this implies that $c_{ijkl}$ are invariants. Hence, the strain energy ${W}_{(G)}$ can be expressed in terms of the invariants

94 $$\begin{aligned} c_{ijkl} \, , {\quad }\lambda _i \, \end{aligned}$$

with the symmetrical properties

95 $$\begin{aligned} c_{ijkl}=c_{jikl}=c_{klij}=c_{ijlk}\, . \end{aligned}$$

Due to the symmetrical property (95), only 24 of the invariants in (94) are independent.These 24 invariants are elements of the irreducible/minimal integrity basis. Hence, all other invariants (see, for example, reference^44^) can be expressed in terms of the 24 independent spectral invariants.

### Infinitesimal strain

The strain energy for a general anisotropic elastic solid is

96 $$\begin{aligned} {W}_{(G)}= & {} {\displaystyle \frac{1}{2}} \left( \text{ tr }[{\mathbb {C}}{\varvec{E}}]{\varvec{E}}\right) = {\displaystyle \frac{1}{2}} \text{ tr }\left( [{\mathbb {C}}({\varvec{E}}^* + {\displaystyle \frac{h}{3}}{\varvec{I}}]({\varvec{E}}^* + {\displaystyle \frac{h}{3}}{\varvec{I}}) \right) \nonumber \\= & {} {\displaystyle \frac{1}{2}} \left( \text{ tr }[{\mathbb {C}}{\varvec{E}}^*]{\varvec{E}}^* + {\displaystyle \frac{2h}{3}} \text{ tr }[{\mathbb {C}}{\varvec{E}}^*]{\varvec{I}}+ {\displaystyle \frac{h^2}{9}} \text{ tr }[{\mathbb {C}}{\varvec{I}}]{\varvec{I}}\right) \, . \end{aligned}$$

The infinitesimal stress takes the form

97 $$\begin{aligned} {\varvec{T}}= {\mathbb {C}}{\varvec{E}}^* + {\displaystyle \frac{h}{3}}{\mathbb {C}}{\varvec{I}}\, . \end{aligned}$$

The hydrostatic stress is

98 $$\begin{aligned} {\displaystyle \frac{\text{ tr }{\varvec{T}}}{3}} = {\displaystyle \frac{\text{ tr }{\mathbb {C}} {\varvec{E}}^*}{3}} + h{\displaystyle \frac{ \text{ tr }{\mathbb {C}}{\varvec{I}}}{9}} \, . \end{aligned}$$

From (98), we have the ground-state bulk modulus

99 $$\begin{aligned} \chi = {\displaystyle \frac{\text{ tr }{\mathbb {C}}{\varvec{I}}}{9}} \, . \end{aligned}$$

In terms of spectral expressions, we have,

100 $$\begin{aligned} {W}_{(G)} = \sum _{i,r=1}^3 \left\{ c_{iirr} \left[ {\displaystyle \frac{e^*_ie^*_r}{2}} + {\displaystyle \frac{he^*_r}{3}} + {\displaystyle \frac{h^2}{18}} \right] \right\} \, , \end{aligned}$$

101 $$\begin{aligned} {\mathbb {C}} = \sum _{i,j,k,l}c_{ijkl} {\varvec{u}}_i\otimes {\varvec{u}}_j\otimes {\varvec{u}}_k\otimes {\varvec{u}}_l \, \end{aligned}$$

and the ground-state bulk modulus

102 $$\begin{aligned} \chi = {\displaystyle \frac{1}{3}}\sum _{i,r=1}^3 c_{iirr} \, . \end{aligned}$$

### Finite strain

In view of (100), we propose a (not the) general finite strain energy for a general anisotropic material, i.e.

103 $$\begin{aligned} {W}_{(G)} = \sum _{i,r=1}^3 c_{iirr} \left( {\displaystyle \frac{f_1(\lambda ^*_i)f_2(\lambda ^*_r)}{2}} + {\displaystyle \frac{g_1(J)f_3(\lambda ^*_r)}{3}} + {\displaystyle \frac{g_2(J)g_3(J)}{18}} \right) + {\phi }_{(I)} \, , \end{aligned}$$

where ${\phi }_{(I)}$ depends on the spectral invariants $\lambda _i$ and $c_{ijkl}$. For example, when specialized to a transversely isotropic material, we have from (71)

104 $$\begin{aligned} {W}_{(G)}= & {} {W}_{(T)} = {\bar{a}}_1 \sum _{i=1}^3 f_1(\lambda ^*_i)f_2(\lambda ^*_i) + {a}_{(2)} g_2(J)g_3(J) + {\bar{a}}_3 \sum _{i=1}^3 \zeta _i f_1(\lambda ^*_i)f_2(\lambda ^*_i) \nonumber \\&+{\bar{a}}_4 \left( \sum _{i=1}^3 \zeta _if_1(\lambda ^*_i)\right) \left( \sum _{i=1}^3 \zeta _if_2(\lambda ^*_i)\right) + {a}_{(5)} g_1(J) \sum _{i=1}^3 \zeta _if_3(\lambda ^*_i) + {\phi }_{(I)} \, . \end{aligned}$$

In this case the spectral components take the form

105 $$\begin{aligned} c_{ijkl}= & {} {\bar{a}}_1(\delta _{ik}\delta _{jl}+\delta _{jk}\delta _{il})+ 2{\bar{a}}_2\delta _{ij}\delta _{kl}+ {\displaystyle \frac{{\bar{a}}_3}{2}}(a_ia_k\delta _{jl} + a_ia_l\delta _{jk} + a_ja_k\delta _{il} + a_ja_l\delta _{ik}) \nonumber \\&+2{\bar{a}}_4 a_ia_ja_ka_l + {\bar{a}}_5(a_ka_l\delta _{ij} + a_ia_j\delta _{kl}) \, . \end{aligned}$$

As mentioned earlier, it is important that ${W}_{(G)}$ satisfies the *P*-property. It is clear that, in view of (92), that the symmetrical part of the *P*-property is satisfied. We now show that ${W}_{(G)}$ has a unique value when two or more of the principal stretches have a same value. Consider the case when $\lambda ^*_1=\lambda ^*_2 =\lambda$. In this case the principal directions ${\varvec{u}}_1$ and ${\varvec{u}}_2$ are not unique but ${\varvec{u}}_3$ has a unique direction. We then have

106 $$\begin{aligned} {W}_{(G)} = W_1 + W_2 + W_3 \, , \end{aligned}$$

where

107 $$\begin{aligned} W_1= & {} {\displaystyle \frac{1}{2}} \left\{ (c_{1111}+c_{2222} + c_{1122}+ c_{2211}) f_1(\lambda )f_2(\lambda ) \right. \nonumber \\&+ \left. (c_{1133}+c_{2233})f_1(\lambda )f_2(\lambda _3) + (c_{3311}+c_{3322})f_1(\lambda _3)f_2(\lambda ) \right\} \, , \end{aligned}$$

108 $$\begin{aligned} W_2= & {} {\displaystyle \frac{g_1(J)}{3}} \left\{ \sum _{i=1}^3 (c_{ii11}+c_{ii22})f_3(\lambda ) + c_{ii33}f_3(\lambda _3) \right\} \, , \end{aligned}$$

109 $$\begin{aligned} W_3= & {} {\displaystyle \frac{1}{18}} \text{ tr }({\mathbb {C}}{\varvec{I}}) g_2(J)g_3(J) \, . \end{aligned}$$

Making use of the relations

110 $$\begin{aligned}&\text{ tr }[{\mathbb {C}}({\varvec{u}}_3\otimes {\varvec{u}}_3)] = c_{1133}+ c_{2233}+c_{3333} =\text{ tr }[({\mathbb {C}}{\varvec{I}})({\varvec{u}}_3\otimes {\varvec{u}}_3)] = c_{3311}+ c_{3322}+c_{3333}, \nonumber \\&\text{ tr }[{\mathbb {C}}{\varvec{I}}] = \sum _{i,r=1}^3 c_{iirr} \, , \end{aligned}$$

we have

111 $$\begin{aligned} W_1= & {} {\displaystyle \frac{1}{2}} \left( Af_1(\lambda )f_2(\lambda ) + B [f_1(\lambda _3)f_2(\lambda ) + f_1(\lambda )f_2(\lambda _3)] \right) \, , \end{aligned}$$

112 $$\begin{aligned} W_2= & {} {\displaystyle \frac{g_1(J)}{3}}\left( (A+B)f_3(\lambda ) + (B+c_{3333})f_3(\lambda _3)\right) \, , \end{aligned}$$

where

113 $$\begin{aligned} A= & {} \text{ tr }[ {\mathbb {C}}{\varvec{I}}- {\mathbb {C}}({\varvec{u}}_3\otimes {\varvec{u}}_3) - {\mathbb {C}}{\varvec{I}}({\varvec{u}}_3\otimes {\varvec{u}}_3) ] + 2c_{3333} \, , {\quad }B = \text{ tr }[{\mathbb {C}}{\varvec{I}}({\varvec{u}}_3\otimes {\varvec{u}}_3)] - c_{3333} \, . \end{aligned}$$

It is clear from (109),(111), (112) and (113) that ${W}_{(G)}$ has a unique value when $\lambda ^*_1=\lambda ^*_2 =\lambda$, since it is independent of the eigenvectors ${\varvec{u}}_1$ and ${\varvec{u}}_2$. Following the above method, it is straightfoward to show that ${W}_{(G)}$ has a unique value when any two of the principal stretches have a same value. In the case when $\lambda ^*_1=\lambda ^*_2 =\lambda ^*_3=\lambda$, the value of ${W}_{(G)}$ is unique since, in this case,

114 $$\begin{aligned} {W}_{(G)} = \text{ tr }[{\mathbb {C}}{\varvec{I}}] \left\{ {\displaystyle \frac{1}{2}}f_1(\lambda )f_2(\lambda ) + {\displaystyle \frac{1}{3}}g_1(J)f_3(\lambda )+ {\displaystyle \frac{1}{18}}g_2(J) g_3(J) \right\} \, . \end{aligned}$$

The weighted Cauchy stress is

115 $$\begin{aligned} J{\varvec{T}}= & {} \sum _{i=1}^3 \left( \lambda ^*_i {\displaystyle \frac{\partial {W}_{(G)}}{\partial \lambda ^*_i}} - p^*\right) {\varvec{v}}_i\otimes {\varvec{v}}_i + \sum _{i\ne j}^3 {\displaystyle \frac{4\lambda ^*_i\lambda ^*_j}{\lambda ^{*2}_i - \lambda ^{*2}_j}}\kappa _{ij} {\varvec{v}}_i\otimes {\varvec{v}}_j \, , \end{aligned}$$

where

116 $$\begin{aligned} \kappa _{ij} = {\displaystyle \frac{\partial {W}_{(G)}}{\partial c_{iiii}}}c_{ijii} - {\displaystyle \frac{\partial {W}_{(G)}}{\partial c_{jjjj}}}c_{ijjj} + \sum _{r\ne i}{\displaystyle \frac{\partial {W}_{(G)}}{\partial c_{iirr}}}c_{ijrr} - \sum _{r\ne j}^3 {\displaystyle \frac{\partial {W}_{(G)}}{\partial c_{jjrr}}}c_{ijrr} \, . \end{aligned}$$

## Example of specific forms of $f_\alpha$ and $g_\alpha$ used in experimental fitting

In this section, we suggest specific forms of the strain functions $f_\alpha$ and $g_\alpha$ to fit experiment data. We strongly emphasize that we are not interested in constructing the (or an) optimal form of $f_\alpha$ and $g_\alpha$ for a particular material; we are only interested in giving examples of specific forms of the proposed strain functions that can be used to fit experimental data. Constructing an optimal form of the strain functions for a particular material similar to the previous work of Shariff^12,14,37^ will be done in the near future. We also emphasize that the specific forms are mainly constructed via visual curve fitting. Since, we are dealing with many types of isotropic and anisotropic materials, curve fitting exercises (such as those found in references^45–47^ for isotropic solids only), for all the isotropic/anisotropic solids mentioned below require a considerable amount of work and analysis, and it is outside the scope of this paper.

Only strain energy functions with ${\phi }_{(I)} =0$ are discussed in this section.

### Isotropic

**Figure 1:**
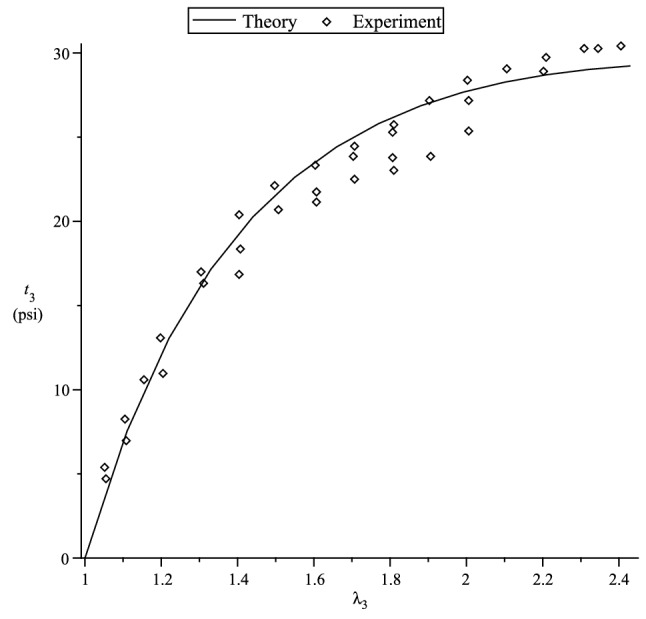
Axial nominal stress $t_3$ versus axial stretch $\lambda _3$. $\mu = 32$ and $\nu =0.25$.

**Figure 2:**
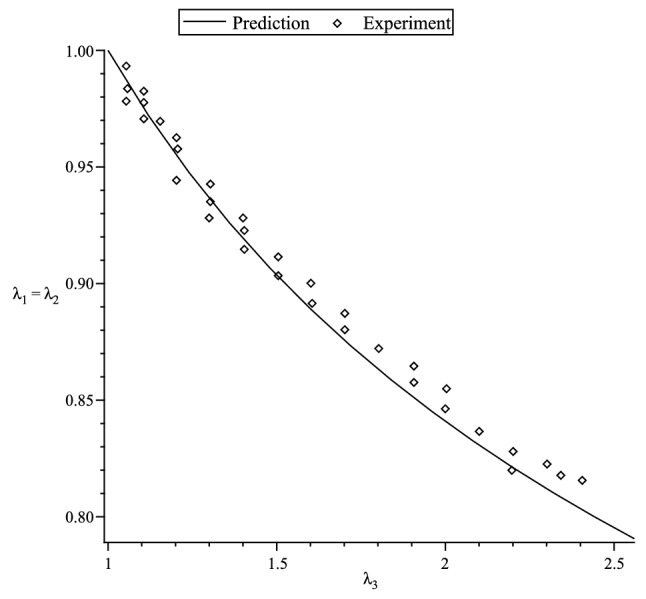
Prediction of the Lateral stretch $\lambda _1=\lambda _2$ versus axial stretch $\lambda _3$.

In the case of a compressible material, we use the simple strain functions based on the Hencky strain energy function^40^

117 $$\begin{aligned} f_1(x)=f_2(x)=g_1(x)=g_2(x) = \ln x \, , \end{aligned}$$

to fit the simple tension data of an isotropic polyurethane foam material used in Blatz and Ko^48^ experiment, where four sets of data are used. The nominal axial stress in the 3-direction is

118 $$\begin{aligned} t_3 = \lambda ^*_3 {\displaystyle \frac{\partial {W}_{(I)}}{\partial \lambda ^*_3}}-\lambda ^*_1 {\displaystyle \frac{\partial {W}_{(I)}}{\partial \lambda ^*_1}} \, . \end{aligned}$$

The values of the lateral stretch $\lambda _1=\lambda _2$ is obtained in terms of $\lambda _3$ via the zero lateral stress condition, i.e.,

119 $$\begin{aligned} \lambda ^*_1 {\displaystyle \frac{\partial {W}_{(I)}}{\partial \lambda ^*_1}} = \lambda ^*_2 {\displaystyle \frac{\partial {W}_{(I)}}{\partial \lambda ^*_2}} \, {\quad }\text{ or } {\quad }\lambda ^*_1 {\displaystyle \frac{\partial {W}_{(I)}}{\partial \lambda ^*_1}} = p^* \, . \end{aligned}$$

We use the ground-state values

120 $$\begin{aligned} \mu = 32 \, \, \text{ psi } \, , {\quad }\nu =0.25 \, \end{aligned}$$

to fit the simple tension data in Fig. 1; these are the same values that are obtained in Blatz and Ko^48^ experiment. Fig. 1 shows that our theory reasonably fit the nominal stress vs. axial stretch curve and the behaviour of the lateral stretch $\lambda _1=\lambda _2$ in terms of the axial stretch $\lambda _3$ is *predicted* quite well in Fig. 2.

**Figure 3:**
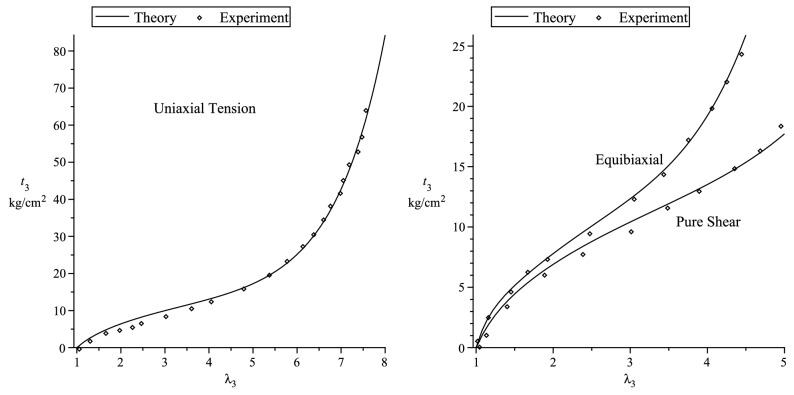
Comparison of theoretical curves with the Treloar’s^49^ experimental data.

**Figure 4:**
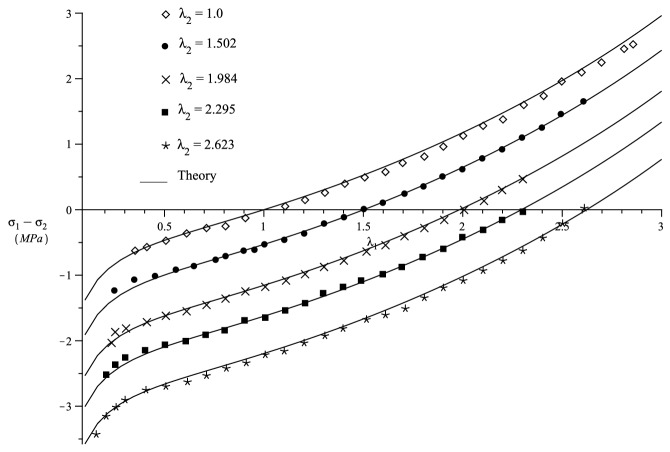
Comparison of theory with the biaxial experimental data of Jones and Treloar^50^.

For incompressible materials ($J=1$), we give, below, specific forms for the strain functions to fit the experimental data of Treloar^49^ and Jones and Treloar^50^. In the case of Treloar’s^49^ data, we use the strain functions^37^

121 $$\begin{aligned} f_1= & {} f_2 \, , {\quad }f_1^2(x) = r(x) = \ln (x)^2 + 3(1.37334\psi _1(x)+ 0.471163\times 10^{-1}\psi _2 \nonumber \\&+0.841383\times 10^{-4}\psi _3) \ge 0 \, \end{aligned}$$

to fit Treloar^49^ experimental data, where

122 $$\begin{aligned} \psi _1 = \int _1^x {\displaystyle \frac{e^{1-s}}{s}} \, ds + x-2\ln x -1\, , {\quad }\psi _2 = \int _1^x {\displaystyle \frac{e^{s-1}}{s}} - x + 1 \, , {\quad }\psi _3 = \int _1^x {\displaystyle \frac{(s-1)^3}{s^{4.6}}} \, ds \, . \end{aligned}$$

In uniaxial, pure shear and equibiaxial extensions, the principal nominal stress $t_3$ can be simply expressed as

123 $$\begin{aligned} t_3 = {\displaystyle \frac{\lambda _3r'(\lambda _3) - {\displaystyle \frac{1}{\lambda _3^m}}r'\left({\displaystyle \frac{1}{\lambda _3^m}}\right)}{\lambda _3}} \, , \end{aligned}$$

where $m=0.5,1,2$ corresponds, respectively, to uniaxial, pure shear and equibiaxial deformatons. It is clear in Fig. 3, that the theoretical curves for the three different deformations fit the experiment data very well when the shear modulus has the value ${\displaystyle \mu = {\displaystyle \frac{11.009}{3}} }$ kg/$\hbox {cm}^2$.

To fit Jones and Treloar^50^ biaxial experimental data, we use $\mu =0.4$(MPa) and the strain functions

124 $$\begin{aligned} f_1 = f_2 \, , {\quad }f_1^2(x) = r(x) = \ln (x)^2 + 2.4669\psi _1(x)+ 0.3771\psi _2 \ge 0\, . \end{aligned}$$

The biaxial deformation experiment require the stress difference

125 $$\begin{aligned} \sigma _1 - \sigma _2 = \mu (\lambda _1r'(\lambda _1) - \lambda _2r'(\lambda _2)) \, , \end{aligned}$$

where $\sigma _1$ and $\sigma _2$ are principal Cauchy stresses. Fig. 4 indicates that the strain functions (124) fits the experiment extremely well.

### Transversely isotropic

**Figure 5:**
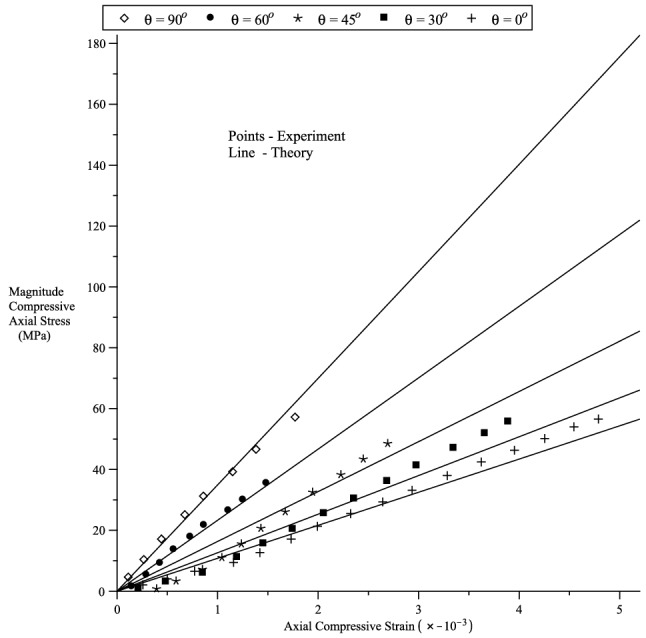
Stress–strain curves for a uniaxial compression deformation. The points are from the experimental test of Jin et al.^51^.

We compare our theory with the axial compression experiment of Jin et al.^51^ on compressible rectangular slabs of transversely isotropic Marcellus shale. Although the measured experimental strains are infinitesimal, the stress-strain behaviour is very mildly nonlinear^42^. Since, the strains are infinitesimal, the Cauchy and nominal stress are indistinguishable. The nominal stress-strain relation

126 $$\begin{aligned} t_3 = {\displaystyle \frac{\lambda ^*_3 {\displaystyle \frac{\partial W}{\partial \lambda ^*_3}} - p^*}{\lambda _3}} \end{aligned}$$

is required for the curve fitting and the rock is compressed in the 3-direction. In general, $\lambda _1\ne \lambda _2$ and their values are obtained via the relations

127 $$\begin{aligned} \lambda ^*_1{\displaystyle \frac{\partial W}{\partial \lambda ^*_1}} = p^* \, , {\quad }\lambda ^*_2{\displaystyle \frac{\partial W}{\partial \lambda ^*_2}} = p^* \, \end{aligned}$$

that correspond to the lateral stress-free condition. To compare our theory with the experiment of Jin et al.^51^, we consider the preferred direction

128 $$\begin{aligned} {\varvec{a}}= \cos \theta {\varvec{g}}_3 + \sin \theta {\varvec{g}}_2 \, , \end{aligned}$$

where $\{{\varvec{g}}_1,{\varvec{g}}_2,{\varvec{g}}_3 \}$ is the Cartesian basis and $\theta$ is the angle ${\varvec{a}}$ makes with the direction ${\varvec{g}}_3$. The simple strain functions

129 $$\begin{aligned} f_\alpha (x) = g_1(x)=g_2(x)=g_3(x) = \ln (x) \, , {\quad }\alpha =1,2,\ldots 7 \, \end{aligned}$$

are used in the curve fitting. The values of the ground-state constants used are

130 $$\begin{aligned} E_a= & {} 75.146\text{ GPa } \, ,{\quad }E_p=34.614\, \text{ GPa } \, ,\nonumber \\ \nu _{zp}= & {} 0.154 \, ,{\quad }\nu _p = 0.374\, ,{\quad }\mu _a =5.48 \text{ GPa }. \end{aligned}$$

We note that Jin et al.^51^, experimentally, obtained the ground-state constant values:

131 $$\begin{aligned} E_a= & {} 16.12 \pm 1.29 \, \text{ GPa } \, ,{\quad }E_p=37.72 \pm 7.04\, \text{ GPa } \, ,\nonumber \\ \nu _{zp}= & {} 0.35 \pm 0.15 \, ,{\quad }\nu _p =0.25 \pm 0.01 \, ,{\quad }\mu _a =6.87 \pm 1.19 \, \text{ GPa }. \end{aligned}$$

However, we are not able to satisfactorily fit the data using the data values in (131), but some of our values are quite close to the values obtained by Jin et al.^51^.

We plot the strain-stress equation (126) when ${\varvec{a}}$ has the directions described by the angles $\theta =0^o,30^o,45^o,60^o,90^o$. The compression curves are given in Fig. 5 and it is clear that our theory capture the behaviour of the experimental data.

**Figure 6:**
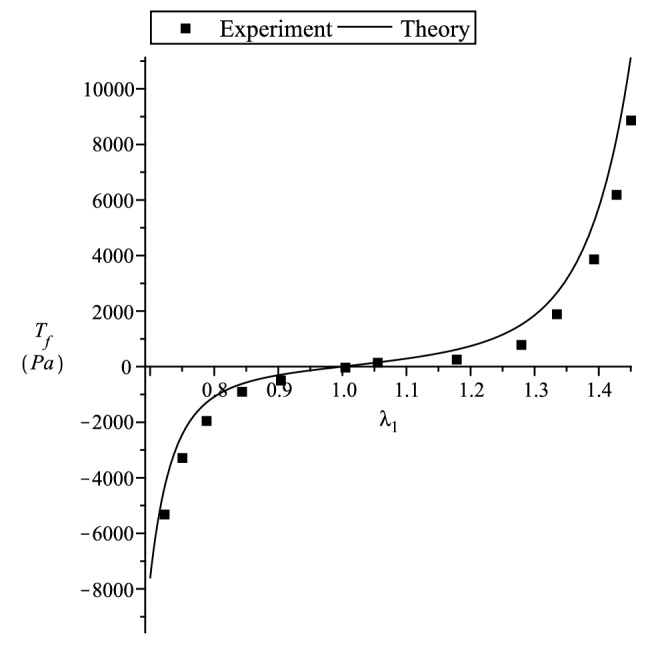
Fitting Chui et al.^52^ porcine liver uniaxial deformation in the fibre direction. $\mu _T=200$ Pa, $\mu _L=600/550$ Pa, $\beta =0$ Pa, $\alpha _1=\alpha _2=\alpha _3=5.7$.

**Figure 7:**
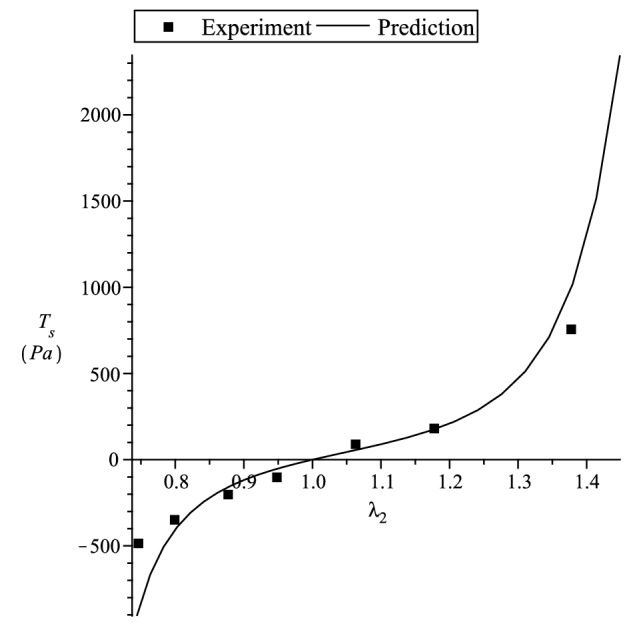
Predicting Chui et al.^52^ porcine liver uniaxial deformation in transverse direction. $\mu _T=200$ Pa, $\mu _L=600/550$ Pa, $\beta =0$ Pa, $\alpha _1=\alpha _2=\alpha _3=5.7$.

**Figure 8:**
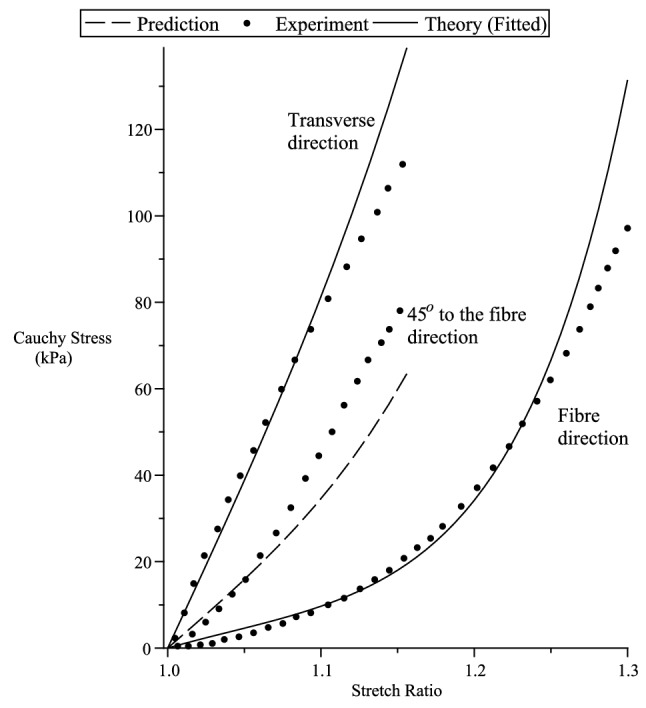
Takaza et al.^53^ uniaxial experiment. $\mu _T=200$ kPa, $\mu _L=70$ Pa, $\beta =0$ kPa, $\alpha _1=5$, $\alpha _2=\alpha _3=3.2$.

**Figure 9:**
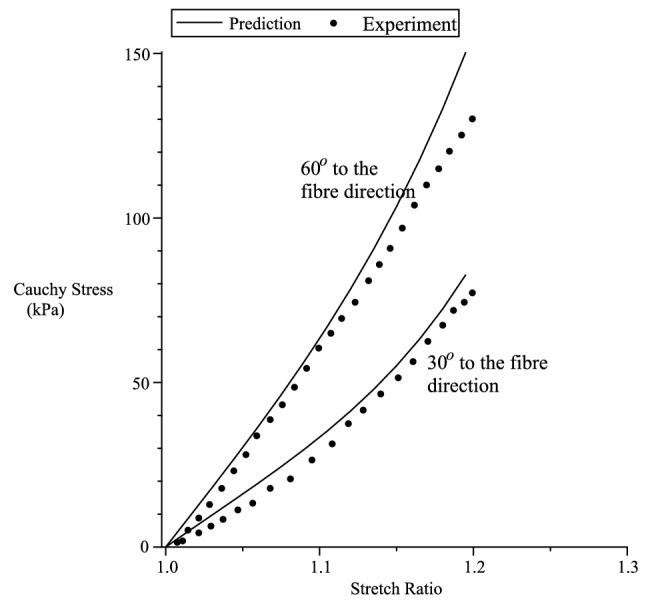
Predicting Takaza et al.^53^ uniaxial experiment. $\mu _T=200$ kPa, $\mu _L=70$ kPa, $\beta =0$ kPa, $\alpha _1=5,\alpha _2=\alpha _3=3.2$.

To compare with the experimental data given in this section for incompressible materials, we consider a strain energy function of the form^14^

132 $$\begin{aligned} {W}_{(T)} =\sum _{i=1}^3 \left[ \mu _T f_1^2(\lambda _i) + 2(\mu _L(I_4)-\mu _T)\zeta _if_2^2(\lambda _i) \right] + {\displaystyle \frac{\beta }{2}}(I_4)\left( \sum _{i=1}^3 \zeta _if_3(\lambda _i) \right) ^2 \, , \end{aligned}$$

where the constants $\mu _T$ and $\mu _L$ , represent the elastic shear moduli in the ground state. The other ground state elastic constant $\beta$^41^ can be related to an elastic constant which has more direct physical interpretations, such as the extension moduli. Since the ground-state constant values when the fibre tension is different from when fibre compression (see Appendix A in Supplementary information), we have,

133 $$\begin{aligned} \mu _L(I_4) = l_p {q}_{(p)}(I_4) + l_n {q}_{(n)}(I_4) \, , {\quad }\beta (I_4) = m_p {q}_{(p)}(I_4) + m_n {q}_{(n)}(I_4) \, , {\quad }I_4={\varvec{a}}\cdot {\varvec{C}}{\varvec{a}}\, . \end{aligned}$$

In this section we compare our theory with the uniaxial tension and compression experiment of Chui et al.^52^ and Takaza et al.^53^ multiple angle uniaxial experiment on soft tissue. We note that in Chui et al.^52^ the uniaxial stretch in the fibre direction is the stiffest, where else Takaza et al.^53^ experiment indicates that the transverse stress is the stiffest. In soft tissues, the initial large extension is generally achieved at relatively low levels of stress with subsequent stiffening at higher levels of extension. This behaviour is due to the recruitment of collagen fibres as they become uncrimped and reach their natural lengths. The inverse error function $erf^{-1}(x)$ seems a good candidate to describe the above mentioned soft tissue stress-strain behaviour since it has low initial gradients followed by high gradients at higher values of *x*. In view of this, for simplicity, to fit the experiments, we use the strain functions

134 $$\begin{aligned} f_1^2(x)= & {} \int _1^x {\displaystyle \frac{4}{\alpha _1\sqrt{\pi }}}erf^{-1}(\alpha _1ln(y)) \, dy \ge 0 \, , {\quad }f_2^2(x)= \int _1^x {\displaystyle \frac{4}{\alpha _2\sqrt{\pi }}}erf^{-1}(\alpha _2ln(y)) \, dy \, \ge 0 \, ,\nonumber \\ f_3(x)= & {} {\displaystyle \frac{2}{\alpha _3\sqrt{\pi }}}erf^{-1}(\alpha _3ln(x)) \, , \end{aligned}$$

where $\alpha _{1-3} \ne 0$ are dimensionless material parameters.

The tensor and vector components used below are with respect to the Cartesian basis $\{{\varvec{g}}_1,{\varvec{g}}_2,{\varvec{g}}_3 \}$. The stress-strain relations are based on that given in (13). We first consider Chui et al.^52^ uniaxial tension and compression experiment. The nominal stresses ${\displaystyle T_f={\displaystyle \frac{t_{11}}{\lambda _1}}}$ for ${\varvec{a}}\equiv [1,0,0]^T$ and ${\displaystyle T_t={\displaystyle \frac{t_{11}}{\lambda _1}}}$ for ${\varvec{a}}\equiv [0,1,0]^T$ are plotted. We use $\mu _T=200$, $\beta =0$ and for $\mu _L$, $l_p=600$ and $l_n=550$. In Fig. 6 we curve fit the data. Using the curve fitted material constant values, in Fig. 7 we predict the experimental data for the transverse stress. It is clear from Fig. 7 that our theory predicts the data quite well.

In the case of Takaza et al.^53^ experiment, we plot the Cauchy stress components $t_{11}$ vs $\lambda _1$. In Fig. 8 we curve fit for ${\varvec{a}}\equiv [1,0,0]^T$ (fibre direction stress) and ${\varvec{a}}\equiv [0,1,0]^T$ (transverse direction stress). Using the fitted numerical values for the material constants, we predict the stress for ${\varvec{a}}\equiv [cos(45^o),sin(45^o),0]^T$ (shown in Fig. 8) and, ${\varvec{a}}\equiv [cos(30^o),sin(30^o),0]^T$ and ${\varvec{a}}\equiv [cos(60^o),sin(60^o),0]^T$, both shown in Fig. 9. The values for $\mu _L$ are $l_p=70$ and $l_n=0$. For simplicity, we have assumed, on the onset, $\beta =0$ and $\alpha _2=\alpha _3$.

### Orthotropic

**Figure 10:**
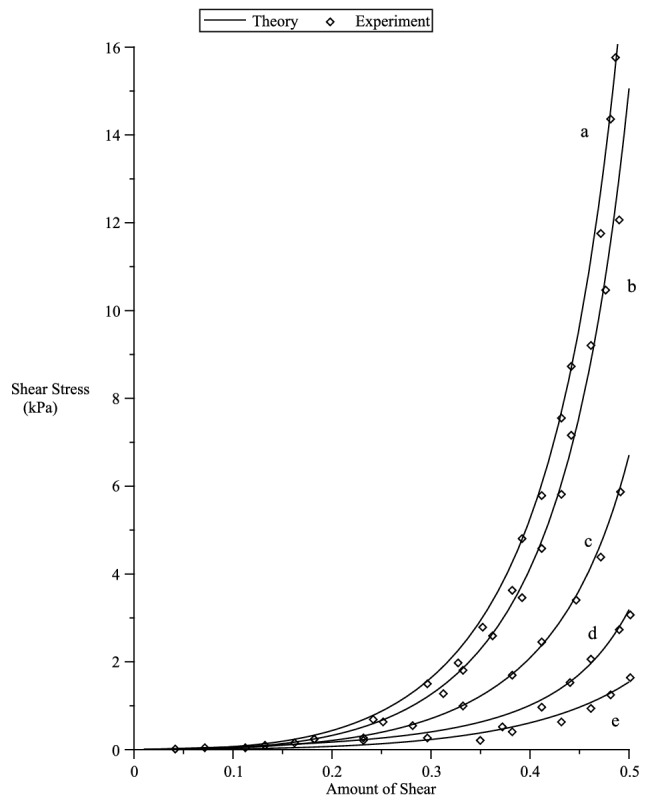
Fitting of our theory with the experiment of Dokos et al.^54^ simple shear data in various sheet/fibre directions. $\rho _0=3.416,\rho _1=0.437, {n}_{(1)}=0.353, {n}_{(2)}=-0.534, {n}_{(3)}=0.218, {n}_{(5)}=47.612, {n}_{(6)}=4.3865, {n}_{(7)}=-25.$.

**Figure 11:**
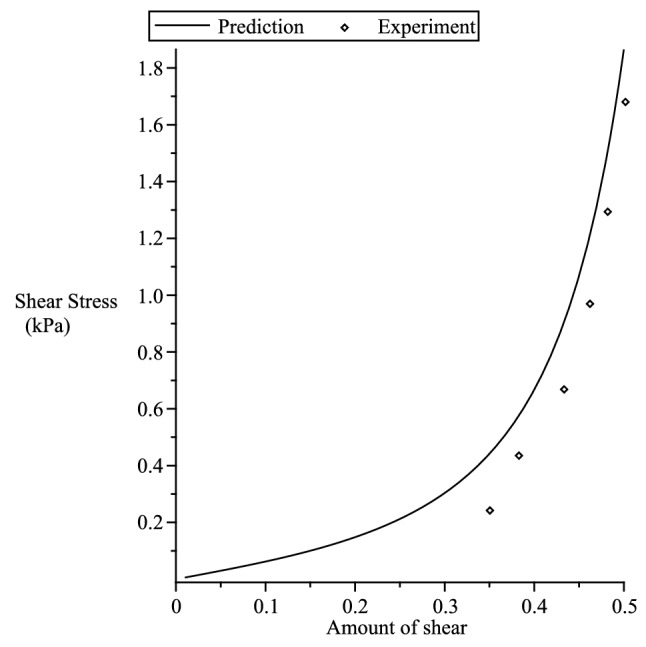
Prediction of fibre/sheet($[0,0,1]^T/[1,0,0]^T$) directions of the simple shear Dokos et al.^54^ experiment. $\rho _0=3.416,\rho _1=0.437, {n}_{(1)}=0.353, {n}_{(2)}=-0.534, {n}_{(3)}=0.218, {n}_{(5)}=47.612, {n}_{(6)}=4.3865, {n}_{(7)}=-25$.

Here, we only consider fitting our theory with the incompressible simple shear experimental data of Dokos et al.^54^ on passive myocardium, where the material can be considered to be orthotropic^55^. We consider the strain energy^12^

135 $$\begin{aligned} {W}_{(O)}= & {} \sum _{i=1}^3 \left[ {n}_{(1)}s^2(\lambda _i) + {n}_{(2)}\zeta _is^2(\lambda _i) + {n}_{(3)}\gamma _is^2(\lambda _i) \right] + {n}_{(5)}\left( \sum _{i=1}^3\zeta _is(\lambda _i)\right) ^2 \nonumber \\&+ {n}_{(6)}\left( \sum _{i=1}^3\gamma _is(\lambda _i)\right) ^2 + {n}_{(7)}\sum _{i=1}^3\zeta _is(\lambda _i)\sum _{i=1}^3\gamma _is(\lambda _i) \, , \end{aligned}$$

where the strain function

136 $$\begin{aligned} s(x) = {\displaystyle \frac{2}{\rho _0\sqrt{\pi }}}erf^{-1}(\rho _0ln(x)) + \rho _1(e^{1-x} + x-2) \, , \end{aligned}$$

$erf^{-1}(x)$ is the inverse error function and, $\rho _o$ and $\rho _1$ are dimensionless material parameters. The tensor and vector components given below are with respect to the Cartesian basis $\{{\varvec{g}}_1,{\varvec{g}}_2,{\varvec{g}}_3 \}$. Following the work of Shariff^12^ the shear stress is given by

137 $$\begin{aligned} \sigma _{12} = 2\left[ l_1(\gamma s^2 + cs) + l_2(\gamma c^2 -cs) + l_3\gamma cs \right] \, , \end{aligned}$$

where $\gamma$ is the amount of shear,

138 $$\begin{aligned} l_1= & {} {\displaystyle \frac{1}{2\lambda _1}}{\displaystyle \frac{\partial {W}_{(O)}}{\partial \lambda _1}} \, , {\quad }l_2={\displaystyle \frac{1}{2\lambda _2}}{\displaystyle \frac{\partial {W}_{(O)}}{\partial \lambda _2}} \, , \end{aligned}$$

139 $$\begin{aligned} l_3= & {} {\displaystyle \frac{1}{\lambda _1^2 -\lambda _2^2}} \left[ \left( {\displaystyle \frac{\partial {W}_{(O)}}{\partial \zeta _1}} - {\displaystyle \frac{\partial {W}_{(O)}}{\partial \zeta _2}}\right) a_1a_2 + \left( {\displaystyle \frac{\partial {W}_{(O)}}{\partial \gamma _1}} - {\displaystyle \frac{\partial {W}_{(O)}}{\partial \gamma _2}}\right) \iota _1\iota _2 \right] \, , \end{aligned}$$

140 $$\begin{aligned} \lambda _1= & {} {\displaystyle \frac{\gamma + \sqrt{\gamma ^2+4}}{2}} \ge 1 , {\quad }\lambda _2={\displaystyle \frac{1}{\lambda _1}} = {\displaystyle \frac{\sqrt{\gamma ^2 +4}-\gamma }{2}} \le 1 \, , \end{aligned}$$

and

141 $$\begin{aligned} c = {\displaystyle \frac{1}{\sqrt{1+\lambda _1^2}}} \, , {\quad }s= {\displaystyle \frac{\lambda _1}{\sqrt{1+\lambda _1^2}}} \, . \end{aligned}$$

Let ${\varvec{a}}$ and ${\varvec{b}}$ represent the fibre and sheet directions^55^, respectively, of the passive myocardium.

In Figure 10, there are six sets of data, however, the experimental data corresponding to the fibre/sheet directions of the passive myocardium with Cartesian components $[1,0,0]^T/[0,0,1]^T$ and $[0,0,1]^T$ $/[1,0,0]^T$ are indistinguishable. We note that no experiment is perfect. This indistinguishable behaviour could be caused by minute errors or approximations in the experiment or it could be the actual behaviour of the myocardium specimen or other unknown factors.

We first fit the five sets of data that correspond to fibre/sheet directions with Cartesian components (from top to bottom in Figure 10) : (a) $[0,1,0]^T/[1,0,0]^T$ (b) $[0,1,0]^T/[0,0,1]^T$ (c) $[1,0,0]^T/[0,1,0]^T$ (d) $[0,0,1]^T/[0,1,0]^T$ (e)$[1,0,0]^T/[0,0,1]^T$. It is clear in Figure 10 that very good agreement is indicated between the model and the experimental data.

Using the fitted values, we then predict the set of data that corresponds to the fibre/sheet directions with components $[0,0,1]^T/[1,0,0]^T$. The predicted curve shown in Fig. 11 is also in good agreement with the experimental data.

## Finite element implementation

In order to obtain numerical solutions for nonlinear isotropic and anisotropic elastic problems, a finite element software, such as Abaqus^56^, requires the end users to supply an explicit expression for the consistent tangent modulus tensor for an invariant-based potential function. In many cases, the consistent (algorithmic) tangent modulus tensor

142 $$\begin{aligned} \mathbb {C_T} = {\displaystyle \frac{1}{J}}{\displaystyle \frac{\partial {{\varvec{\tau }}}_{(J)}}{\partial {\varvec{D}}}} \, \end{aligned}$$

is used in the finite element code, where ${{\varvec{\tau }}}_{(J)}$ is the Jaumann rate of the Kirchhoff stress and ${\varvec{D}}$ is the deformation rate tensor. The consistent tangent modulus tensor requires, among others, the explicit expression for the 4th-order tensor (see, for example,^57^)

143 $$\begin{aligned} {\displaystyle \frac{\partial ^2 {W}_{(e)}}{\partial {\varvec{C}}\partial {\varvec{C}}}} \, \, \, , \end{aligned}$$

where ${W}_{(e)}$ represents a strain energy function. Until recently, only consistent tangent modulus tensors for ${W}_{(e)}$ containing “classical” tensor invariants that can be written explicitly in terms of ${\varvec{C}}$ were obtained in the literature; this due to the fact that the second derivative of the classical invariants with respect to ${\varvec{C}}$ (a 4th order tensor) is easily obtained because they can be expressed explicitly in terms of ${\varvec{C}}$. However, the consistent tangent modulus tensor for a potential function ${W}_{(e)}$, containing spectral invariants that cannot be written explicitly in terms of ${\varvec{C}}$ but can be written explicitly in terms of the eigenvalues and eigenvectors of ${\varvec{C}}$, has only recently developed by Shariff^11^ and, in view of this, the spectral formulation of the consistent tangent modulus tensor, developed by Shariff^11^, is not so well-known; hence, it may be mistakenly assumed that the spectral consistent tangent modulus tensor cannot be *explicitly* evaluated and that the proposed model cannot be used in a Finite Element commercial software. So the objective of this section is to report that the spectral consistent tangent modulus tensor can be evaluated using the results given in Shariff^11^.

For isotropic, transversely isotropic and two-preferred direction materials, described above, the strain energy function ${W}_{(e)}$ contains, invariants of the form

144 $$\begin{aligned} I = \sum _{i=1}^3 ({\varvec{u}}_i\cdot {\varvec{G}}{\varvec{u}}_i) g(\lambda ^*_i) {\quad }\text{ and } {\quad }J \, , \end{aligned}$$

where ${\varvec{G}}$ is a second order tensor. In Shariff^11^, the tangent modulus tensor (142) for a strain energy function that contains invariants of the form (144) is explicitly formulated. In the case of the strain energy function ${W}_{(G)}$, given in (103), for a general anisotropic material, the corresponding tangent modulus tensor (142) can be derived using the results given in Shariff^10^; however, due to its complex derivation, we will not derive it here.

## Conclusion

In this contribution, we define a generalized strain function that is similar to the Hill’s^1^ strain function and a volumetric function, where they are used to characterize strain energy functions in isotropic or anisotropic elasticity. These strain functions are single variable functions that depend on an invariant with a clear physical meaning, which facilitates the construction of specific forms of the strain energy function, in the sense that a function of a single variable with a clear physical interpretation is easily manageable and this is indicated in “Example of specific forms of *fα* and *gα* used in experimental fitting” section; they also facilitate the construction of strain energy functions that are consistent with infinitesimal elasticity as described in sections “Isotropic” to “General anisotropy”. The proposed generalized strain functions enable the development of a strain energy function for a general anisotropic material that contains the general 4th order classical stiffness tensor. Having a clear physical interpretation, the spectral invariants are attractive in aiding experiment design. Some previous strain energy functions that appeared in the literature can be considered as special cases of the proposed generalized strain energy functions. The proposed constitutive equations can be easily converted to allow the mechanical influence of compressed fibres to be excluded or partial excluded and to model fibre dispersion in collagenous soft tissues, and they can be easily implemented in a Finite Element software. In “Example of specific forms of *fα* and *gα* used in experimental fitting” section, we show that the suggested crude specific strain function forms fitted well with experimental data and managed to predict several sets of experimental data. The single-variable-function constitutive equations are expected to set a platform for future modelling of various types of anisotropic elastic solids; future modellers only require to construct specific forms of the functions $f_\alpha$ and $g_\alpha$ to model their strain energy functions. The extent of the proposed model applicability to different anisotropic needs to be assessed by comparing it with relevant experimental data of a much wider class of anisotropic materials.

## Supplementary information

Supplementary Information.
