# Supplementary material for: A generalized strain approach to anisotropic elasticity
Source: Sci Rep. 2022 Jan 7;12:172. doi: 10.1038/s41598-021-03842-3 (PMC8742056; doi:10.1038/s41598-021-03842-3)
Supplement: Supplementary file 1 — Supplementary Information. [file 41598_2021_3842_MOESM1_ESM.pdf]

Appendix A

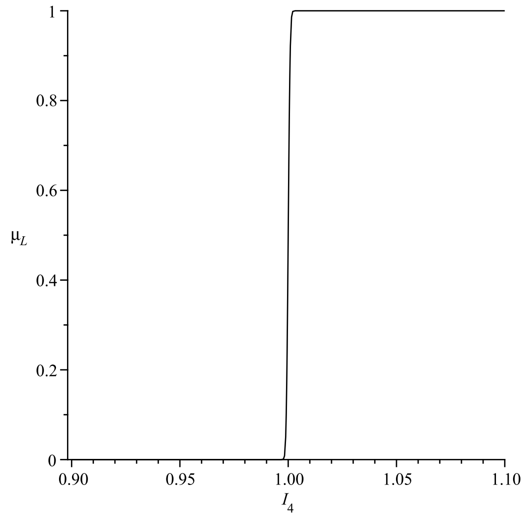

Figure S1: $\mu_L(I_4)$, with $l_p = 1$ and $l_n = 0$

In this Appendix, the modification of the strain energy function to consider fibre compression does not contribute (or partially contribute) towards the strain energy function is exemplified via incompressible transversely isotropic elasticity; this modification has been applied by the author to other types of anisotropic elasticity [44,45]. To take into account that fibre compression does not contribute (or partially contribute) towards the strain energy function, we assume that some of the ground-state constants depend discretely on the invariant $I_4 = \boldsymbol{a} \cdot \boldsymbol{C}\boldsymbol{a}$, i.e.,

$$W_{(T)} = \sum_{i=1}^{3} \left[\mu_T f_1(\lambda_i) f_2(\lambda_i) + 2(\mu_L(I_4) - \mu_T)\zeta_i f_3(\lambda_i) f_4(\lambda_i)\right] + \frac{\beta}{2}(I_4) \left(\sum_{i=1}^{3} \zeta_i f_5(\lambda_i)\right)^2 \tag{A1}$$

for an incompressible transversely isotropic elastic solid [42,50]. However, the discrete functions μ_L and β are difficult to implement, practically. For example, if on the onset we know the numerical value of I_4, then we can easily impose the appropriate numerical values on μ_L and β (or select the appropriate functional form). However, in a general boundary value problem, the value of I_4 depends on the constitutive equation and hence it cannot be used as a hypothesis to decide which of the two discrete forms is required in a boundary value problem solving process. To overcome this "vicious circle", we approximate our proposed discrete constants using the following continuous functions

$$q_{(p)}(x) = \frac{(1 + erf(a(x-1))}{2}, \quad q_{(n)}(x) = \frac{(1 + erf(a(1-x))}{2}, \tag{A2}$$

where erf is the error function and a is very large positive number. We then define

$$\mu_L(I_4) = l_p q_{(p)}(I_4) + l_n q_{(n)}(I_4)\,, \quad \beta(I_4) = m_p q_{(p)}(I_4) + m_n q_{(n)}(I_4)\,, \tag{A3}$$

where the constants l_p and l_n are non-negative [42] and, the constants m_p and m_n can be negative or non-negative, but they must satisfy the stability condition [42]. For the benefit of the readers the plot of $\mu_L(I_4)$ for $l_p = 1$, $l_n = 0$ and $a = 1000$ is given in Fig. S1. It is clear in Fig. S1, that the continuous approximation of the discrete function is not accurate when the argument x (say) of the function is very close to unity. The accuracy of the continuous function could be improve by using a value of a larger that 1000, but in this paper the value $a = 1000$ is sufficient for our purpose. It is important to note that when x is very close to unity the calculated stress is very close to zero and hence the calculated ground state constants do not affect the value of stress significantly for x close to unity. For practical purposes, we let the value of the derivative of all the ground-state constants to be zero.

Appendix B

In this Appendix, as in Appendix A, as an example, we only discuss incompressible transversely isotropic elasticity. The inclusion of the fibre dispersion terms in a two-preferred direction material strain energy function, is given in Shariff and Merodio [44]. The mechanical influence of fibre dispersion in collagenous soft tissues has been studied recently in the literature [10,21]. The collagen fibers in these tissues may be dispersed randomly in space, in a certain pattern such as predominately in a particular direction [55], as a rotationally symmetric dispersion about a mean direction, or as the recently observed non-symmetric dispersion in arterial walls [31]. In this section, we show how the above model can be easily modified to take fibre dispersion into account. Macroscopically, the fibre dispersion model require the dispersion tensor [10]

$$\boldsymbol{H} = k\boldsymbol{I} + (1-3k)\boldsymbol{A}\,, \tag{B1}$$

where the restriction $0 \leq k \leq \dfrac{1}{3}$ is required. Using operations such as

$$\mathrm{tr}\left[(k\boldsymbol{I} + (1-3k)\boldsymbol{A})(\boldsymbol{F}_{(\alpha)}\boldsymbol{F}_{(\beta)})\right] = \sum_{i=1}^{3} \bar{\zeta}_i f_\alpha(\lambda_i) f_\beta(\lambda_i)\,, \tag{B2}$$

where

$$\bar{\zeta}_i = k + (1-3k)\zeta_i\,. \tag{B3}$$

The incompressible constitutive equation then simply takes the form

$$\begin{aligned} W_{(T)} \;&=\; \sum_{i=1}^{3}\left[\mu_T f_1(\lambda_i) f_2(\lambda_i) + 2(\mu_L(I_4) - \mu_T)\bar{\zeta}_i f_3(\lambda_i) f_4(\lambda_i)\right] \\ &+\; \frac{\beta}{2}(I_4)\left(\sum_{i=1}^{3}\bar{\zeta}_i f_5(\lambda_i)\right)^2 . \end{aligned} \tag{B4}$$
